# WDR12, a Member of Nucleolar PeBoW-Complex, Is Up-Regulated in Failing Hearts and Causes Deterioration of Cardiac Function

**DOI:** 10.1371/journal.pone.0124907

**Published:** 2015-04-27

**Authors:** Anne-Mari Moilanen, Jaana Rysä, Leena Kaikkonen, Teemu Karvonen, Erja Mustonen, Raisa Serpi, Zoltán Szabó, Olli Tenhunen, Zsolt Bagyura, Juha Näpänkangas, Pauli Ohukainen, Pasi Tavi, Risto Kerkelä, Margrét Leósdóttir, Björn Wahlstrand, Thomas Hedner, Olle Melander, Heikki Ruskoaho

**Affiliations:** 1 The Institute of Biomedicine, Department of Pharmacology and Toxicology, University of Oulu, Oulu, Finland; 2 Department of Pathology, The Institute of Diagnostics, University of Oulu, Oulu, Finland; 3 Biocenter Oulu, Faculty of Biochemistry and Molecular Medicine, University of Oulu, Oulu, Finland; 4 Heart Center, Semmelweis University, Budapest, Hungary; 5 Department of Biotechnology and Molecular Medicine, A. I. Virtanen Institute for Molecular Sciences, University of Eastern Finland, Kuopio, Finland; 6 Medical Research Center Oulu, Oulu University Hospital and University of Oulu, Oulu, Finland; 7 Institute of Medicine, The Sahlgrenska Academy, University of Gothenburg, Gothenburg, Sweden; 8 Department of Clinical Sciences, Lund University, Lund, Sweden; 9 Department of Internal Medicine, Skåne University Hospital, Malmö, Sweden; 10 Department of Cardiology, Skåne University Hospital, Malmö, Sweden; 11 Division of Pharmacology and Pharmacotherapy, University of Helsinki, Helsinki, Finland; University of Tampere, FINLAND

## Abstract

**Aims:**

In a recent genome-wide association study, WD-repeat domain 12 (WDR12) was associated with early-onset myocardial infarction (MI). However, the function of WDR12 in the heart is unknown.

**Methods and Results:**

We characterized cardiac expression of WDR12, used adenovirus-mediated WDR12 gene delivery to examine effects of WDR12 on left ventricular (LV) remodeling, and analyzed relationship between MI associated WDR12 allele and cardiac function in human subjects. LV WDR12 protein levels were increased in patients with dilated cardiomyopathy and rats post-infarction. In normal adult rat hearts, WDR12 gene delivery into the anterior wall of the LV decreased interventricular septum diastolic and systolic thickness and increased the diastolic and systolic diameters of the LV. Moreover, LV ejection fraction (9.1%, *P*<0.05) and fractional shortening (12.2%, *P*<0.05) were declined. The adverse effects of WDR12 gene delivery on cardiac function were associated with decreased cellular proliferation, activation of p38 mitogen–activated protein kinase (MAPK)/heat shock protein (HSP) 27 pathway, and increased protein levels of Block of proliferation 1 (BOP1), essential for ribosome biogenesis. Post-infarction WDR12 gene delivery decreased E/A ratio (32%, *P*<0.05) suggesting worsening of diastolic function. In human subjects, MI associated WDR12 allele was associated significantly with diastolic dysfunction and left atrial size.

**Conclusions:**

WDR12 triggers distinct deterioration of cardiac function in adult rat heart and the MI associated WDR12 variant is associated with diastolic dysfunction in human subjects.

## Introduction

Heart failure (HF) is one of the most common causes of cardiovascular morbidity and mortality, and its prevalence is rapidly increasing as the mean age of the population advances [1]. Coronary artery disease (CAD) and its main complication, myocardial infarction (MI), is the leading cause of adverse left ventricular (LV) remodeling and HF [2,3]. Recent genome-wide association studies (GWAS) have identified several single nucleotide polymorphisms (SNPs) as reproducibly associated with risk of CAD and MI. In the largest genetic study to date, a total of 46 genomic loci were linked to variation in susceptibility to CAD [4]. Of the 46 genome-wide significant lead SNPs, 12 showed a significant association with a lipid trait and 5 showed significant association with blood pressure. One of the strongest associated variants, located at the WDR12 locus, was initially associated with risk for early-onset MI [5], but its cellular and functional role in the heart is unknown.

WDR proteins contain a series of highly conserved repeat motifs, which typically contains a tryptophan-aspartic acid dipeptide (the WD unit) at its carboxy terminus and a glycine-histidine dipeptide residues downstream from its amino terminus [6]. More than 160 distinct members of WDR proteins have been identified and the majority of them are found in eukaryotes [7]. WDR proteins are associated with several cellular processes such as regulation of signal transduction, pre-mRNA processing, gene transcription, cell cycle progression and cytoskeleton organization [6]. Endogenous WDR12 is crucial for processing of the 32S precursor ribosomal RNA (rRNA) and cell proliferation. High levels of WDR12 mRNA are found in the thymus and testis of adult mice and it is ubiquitously expressed during embryogenesis [8]. A nucleolar complex of endogenous Pescadillo 1 (Pes1), Block of proliferation 1 (BOP1) and WDR12 is termed as PeBoW complex in mammalian cells, and is essential for ribosome biogenesis [9,10].

In the present study, we analyzed the expression of WDR12 in the heart and used WDR12 gene delivery to examine direct functional and structural effects of WDR12 on LV remodeling. Because these experiments revealed that local WDR12 gene delivery deteriorated systolic and diastolic function, we evaluated potential mechanisms triggering the impairment of cardiac function by WDR12 gene overexpression. Furthermore, we analyzed the relationship between MI associated WDR12 allele and cardiac function, measured by echocardiography, in a cohort of approximately 1400 human subjects.

## Materials and Methods

### Ethics Statement

All experimental protocols were approved by the Animal Use and Care Committee of the University of Oulu and the Provincial Government of Western Finland Department of Social Affairs and Health. The investigations conform to the Guiding Principles for Research Involving Animals.

For human studies, the study complies with the Declaration of Helsinki. The study protocol was approved by the Internal Review Committee of the University of Semmelweis, Hungary (Ethical permission number: 5012-0/2011-EKU 142/PI/11–Semmelweis, University; Hungary) and written informed consent was obtained from the subjects, except that the Institution has the ethical permission to collect tissue samples from healthy subjects (victims of accidents).

### Recombinant Adenovirus Vectors

For constructions of rat WDR12, a full-length coding region of WDR12 cDNA was cloned into the *Sal*I and *Hind*III sites of the pShuttle-CMV vector (Qbiogene Inc., Illkirch, Cedex-France). The sequences for the cloning primers used were as follows; WDR12 forward 5'-GCG TCG ACC TTT CTG CCA TGG CAC AAC-3' and reverse 5'-CCC AAG CTT TCA AGC CCC CAC ATG GGA AGT-3'. The pShuttle-CMV-LacZ was a commercial plasmid (Stratagene, La Jolla, CA, USA). Adenoviruses (serotype 5) were prepared by standard protocols (Qbiogene Inc., Illkirch, Cedex-France) and purified by centrifugation on iodixanol (OptiPrep, Axis-Shield PoC AS, Oslo, Norway). The adenoviral titers (infectious unit, ifu) were determined by AdEasy Viral Titer Kit (Stratagene). Infectious unit is biologically equivalent to plaque forming unit (PFU). β -galactosidase concentration was determined by luminescent β -gal Kit (Clontech Laboratories Inc., Palo Alto, CA, USA). The recombinant adenoviruses containing the coding regions of the constitutively active mitogen-activated protein kinase kinases (MKK), MKK3b and MKK6b and wild type p38α, and p38β genes driven by CMV immediate early promoter were generated as described previously [11]. In another series of experiments, we have observed that overexpression of p38α and p38β produce similar p38 and phosho-p38-mitogen–activated protein kinase (MAPK) levels in cardiomyocytes (Kaikkonen *et al*., unpublished observation). MKK and p38 adenoviruses were generously supplied by Dr. Veli-Matti Kähäri from the University of Turku, Finland.

### Intramyocardial Gene Transfer

We and others have previously shown that local injection of adenoviral constructs into the left ventricular free wall is an efficient site-specific method of gene delivery that targets high expression of the transgene in the LV without affecting other organs or other regions of the heart [12,13]. Male Sprague-Dawley rats weighing 250–300 g were anesthetized with medetomidine hydrochloride (Domitor, 250 μg/kg i.p.) and ketamine hydrochloride (Ketamine, 50 mg/kg i.p.). Rats were connected to the respirator through a tracheotomy. A left thoracotomy and pericardial incision was performed. Adenovirus-mediated gene transfer into the LV free wall was performed as previously described [13,14]. Different doses of adenoviral constructs were first tested to increase LV WDR12 protein levels. Then, recombinant adenovirus (4 x 10^9^ ifu), in a 100 μl volume, was injected using a Hamilton precision syringe directly into the anterior wall of the LV. The syringe was inserted in one site of the LV free wall (apex to base), and then slowly the solution was injected while withdrawing the syringe. After the operation, anaesthesia was partially antagonized with atipamezole hydrochloride (Antisedan, 1.5 mg/kg i.p.) and rats were hydrated with physiological saline solution (5 ml s.c.). For postoperative analgesia, buprenorphine hydrochloride (Vetergesic, 0.05–0.2 mg/kg s.c.) was administered. The total number of animals used was 240.

### Acute Myocardial Infarction

MI was produced by ligation of the left anterior descending coronary artery (LAD) as previously described [13,15]. The sham-operated rats underwent the same surgical procedure without the ligation of LAD. Recombinant adenovirus was injected into the anterior wall of the LV before the ligation of LAD. The adenoviral gene delivery to the sham-operated hearts was performed using the same technique without the ligation of LAD.

### Angiotensin II-Mediated Hypertension

Angiotensin II (Ang II, 33.3 μg/kg/h) (a generous gift from Dr. Olli Vuolteenaho, University of Oulu, Finland), was administered via subcutaneously implanted osmotic minipumps for 6 hours and 2 weeks (Alzet, Scanbur BK AB, Sollentuna, Sweden), as described previously [16,17]. Minipumps were implanted subcutaneously before the gene delivery. Ang II–mediated hypertension has been used extensively in key studies in the development of antihypertensive agents [18–21]. Using this experimental model of hypertension, mean arterial pressure increases rapidly (within 3 hours) and remains significantly elevated throughout the 2-weeks period [16]. Under our experimental conditions, Ang II type 1 receptor (AT_1_-R) blockade by losartan completely abolished Ang II–induced changes in the cardiac gene expression, left ventricular weight to body weight ratio and hemodynamics [17].

### Echocardiographic Measurements

Transthoracic echocardiography was performed using the Acuson Ultrasound System (Sequoia 512) and a 15-MHz linear transducer (15L8) (Acuson, MountainView, CA, USA) as previously described [13,22]. Before examination, rats were sedated with ketamine (50 mg/kg i.p.) and xylazine (10 mg/kg i.p.). Using two-dimensional imaging, a short axis view of the LV at the level of the papillary muscles was obtained, and a two dimensionally guided M-mode recording through the anterior and posterior walls of the LV was obtained. LV end-systolic and end-diastolic dimensions as well as the thickness of the interventricular septum and posterior wall were measured from the M-mode tracings. LV fractional shortening (FS) and ejection fraction (LVEF) were calculated from the M-mode LV dimensions using the Eqs 1 and 2:

$$\text{FS}\;(\%)=\{\frac{\left(LVEDD-LVESD\right)}{LVEDD}\}\times 100$$ (1)

$$\text{LVEF}\;(\%)=\{\frac{{\left(LVEDD\right)}^{3}-{\left(LVESD\right)}^{3}}{LVEDD^{3}}\}\times 100$$ (2)

An average of three measurements of each variable was used. All echocardiographical measurements were performed blinded by persons (E.M. and Z.Sz.), who were not aware of the treatments. After echocardiography, the animals were sacrificed. Hearts were weighed and the ventricles were immersed in liquid nitrogen and stored at—70°C for later analysis.

### Extraction of Cytoplasmic Protein and Western Blot Analyses

To extract the cytoplasmic protein, the left ventricular tissue was broken and reduced to a powder in liquid nitrogen. The thawed powder was homogenized in a lysis buffer (20 mmol/l Tris-HCl (pH 7.5), 10 mmol/l NaCl, 0.1 mmol/l EDTA, 0.1 mmol/l EGTA, 1 mmol/l β-glycerophosphate, 1 mmol/l Na_3_VO_4_, 2 mmol/l benzamidine, 1 mmol/l phenylmethylsulfoxide, 50 mmol/l NaF, 1 mmol/l dithiothreitol and 10 μg/ml each of leupeptin, pepstatin and aprotinin). The cytosolic fraction was separated out by centrifugation at 2000 rpm in +4°C for 1 minute. To separate the cytoplasmic protein fraction, 5 x nuclear extraction buffer (NEB) (100 mM Tris-HCl (pH 7.5), 750 mM NaCl, 5 mM EDTA, 5 mM EGTA, 5% Triton X 100, 12 mM sodium pyrophosphate, 5 mM β-glycerophosphate, 5 mM Na_3_VO_4_) was added to the tissue homogenate following by centrifugation at 12500 rpm in +4°C for 20 minutes. The supernatant was frozen in liquid nitrogen and stored in—70°C until assayed.

To extract the nuclear protein fraction, the supernatant from the first centrifugation was incubated on ice for 15 minutes, NP-40 was added, and the nuclei were collected by centrifugation at 12500 rpm for 30 seconds. The pellet was resuspended in a solution containing 20 mM HEPES (pH 7.9), 0.4 M NaCl, 1 mM EDTA, 1mM EGTA, 1 mM Na_3_VO_4_, 2 mM benzamidine, 1 mM PMSF, 50 mM NaF, 1 mM DTT, 3 μg/ml 1-chloro-3-tosylamido-7-phenyl-2-butanone (TPCK), 3 μg/ml L-1-tosylamido-2-phenylethyl chloromethyl ketone (TLCK), and 10 μg/ml each of leupeptin, pepstatin and aprotinin. The samples were incubated at +4°C for 30 minutes, centrifuged at 12500 rpm for 5 minutes and the resulting supernatants frozen in liquid nitrogen and stored in—70°C until assayed. All protein concentrations were determined by Bio-Rad protein assay.

In the western blot analysis, 30 μg total protein or nuclear protein was subjected to SDS-PAGE and the separated proteins were electrically transferred to Optitran BA-S 85 nitrocellulose membranes (Scleicher & Schuell BioScience, Dassel, Germany). After blocking the nonspecific background in a mixture (1:1) of Odyssey Blocking Buffer—Tris–buffered saline (TBS), nitrocellulose membranes were incubated with appropriate primary antibody in Odyssey Blocking Buffer–TBS overnight at +4°C. Nitrocellulose membranes were incubated with antiphospho-p38-MAPK, antiphospho-ERK1/2, antisarcoplasmic reticulum Ca^2+^-ATPase (SERCA2), anti-p38-MAPK or anti-ERK1/2, anti-WDR12, anti-BOP1, anti-Pescadillo, anti-phospho-heat shock protein 27 (HSP27), anti-HSP27, anti-Lamin B and anti-GAPDH-antibody. The antibody dilution varied from 1:200 to 1:2000, depending on the signal strength. After washing, antibody binding was detected with specific secondary antibodies, fluorescent (Alexa Fluor) antibodies (1:3000–1:5000) used with Odyssey Infrared Imaging System.

For a second western blot, the membranes were stripped in buffer containing 62.5 mmol/l tris (pH 6.8), 2% sodium dodecyl sulfate, and 100 mmol/l mercapthoethanol. The detection was based on fluorescence measured by the Odyssey Infrared Imaging System. The bands were scanned and analyzed with Quantity One software (Bio-Rad Laboratories, Hercules, CA, USA). Antibodies were obtained from Millipore (Temecula, CA, USA), Invitrogen (Carlsbad, CA, USA), Aviva systems biology (San Diego, CA, USA), Santa Cruz Biotechnology (CA, USA) and Cell Signaling Technology (Beverly, MA, USA).

### Isolation and Analysis of RNA

The RNA was extracted from the left ventricular tissue by using the guanidine-thiocyanate-CsCl method [23]. Cyclin-dependent kinase 4 inhibitor 2c (Cdkn2c), p19ARFexon1b, p16INK4a, cyclin-dependent kinase 4 inhibitor 2b (Cdkn2b), tropomyosin 1, WDR12, fibronectin-1 and 18S mRNA levels were analyzed by the RT-PCR using TaqMan chemistry on an ABI 7300 Sequence Detection System (Applied Biosystems) as previously described [24–26]. The sequences of the forward and reverse primers and for fluorogenic probes for RNA detection are shown in Table 1. The results were normalized to 18S RNA quantified from the same samples.

**Table 1:** Forward and reverse primer and fluorogenic probe sequences used for real time quantitative RT-PCR analysis.

Gene		Sequence
BNP	Forward	TGGGCAGAAGATAGACCGGA
	Reverse	ACAACCTCAGCCCGTCACAG
	Fluorogenic probe	CGGCGCAGTCAGTCGCTTGG
ANP	Forward	GAAAAGCAAACTGAGGGCTCTG
	Reverse	CCTACCCCCGAAGCAGCT
	Fluorogenic probe	TCGCTGGCCCTCGGAGCCT
Fibronectin-1	Forward	GCGAGGCAGGATCAGCTG
	Reverse	CCAATCTTGTAGGACTGACCCC
	Fluorogenic probe	ACCATTGCAAATCGCTGCCATGAA
Tpm1	Forward	GAAAGCCGAGCCCAAAAAG
	Reverse	GCTTACGGGCCACCTCTTC
	Fluorogenic probe	TCCAGCTGAAAGAGGCCAAGCACATT
p19ARFexon1b	Forward	GGTGCAGTTCCTGGGATCCT
	Reverse	TCAACACCAAGGCCACGAA
	Fluorogenic probe	CGACCCAGGTCAGCGAACGGC
p16INK4a	Forward	TCCTCCGCTGGGAACGT
	Reverse	GGCGTGCTTGAGCAGAAGTT
	Fluorogenic probe	TCCCGGGTCACCGACAGGCA
Cdkn2b	Forward	CCCTCACCAGACCTGTGCAT
	Reverse	CAGGCGTCACACACATCCA
	Fluorogenic probe	CTTCCTGGACACGCTAATGGTGCTGC
Cdkn2c	Forward	GAGGTGCTAATCCCAATTTGAAA
	Reverse	AGCCTGTACAGTGTCCAGGAAAC
	Fluorogenic probe	ACCGAACTGGTTTTGCTGTCATTCACG
WDR12	Forward	TGGCTTGGGTGAAAAAAGACA
	Reverse	TCCCATAAGAGAATAGTCTGGTCCAT
	Fluorogenic probe	TCTGTCTTGCCTACTCCTGACGGCCTC
18S	Forward	TGGTTGCAAAGCTGAAACTTAAAG
	Reverse	AGTCAAATTAAGCCGCAGGC
	Fluorogenic probe	CCTGGTGGTGCCCTTCCGTCA

BNP, B-type natriuretic peptide; ANP, atrial natriuretic peptide; Cdkn2b, cyclin-dependent kinase 4 inhibitor 2b; Cdkn2c, cyclin-dependent kinase 4 inhibitor 2c; Tpm1, Tropomyosin 1; WDR12, WD-repeat domain 12; TGFβ1, transforming growth factor β1; RT-PCR, real time quantitative reverse transcription-PCR.

### Histology, Immunohistochemistry and Image Analysis

For histological analysis, the LVs were fixed in 10% buffered formalin solution. Transversal sections of the LV were embedded in paraffin, and 5-μm sections were cut. Sections were cut from the mid-section of the heart, at the level of the papillary muscles. Samples from different animals were obtained in an identical way and from the corresponding sites in order to make the samples fully comparable. Sections were stained with hematoxylin and eosin or Massons's trichrome to examine the fibrotic area of the LV. Previously we have studied the local response to adenovirus-mediated gene transfer by measuring the fibrotic area in the LV at 2 weeks after intramyocardial injection of adenoviral construct expressing LacZ and PBS-based buffer (3%-iodixanol-PBS) as well as from the hearts with needle-stick (no injection of fluid) and non-injected hearts. The results demonstrated that the degree of fibrosis did not differ between PBS-based buffer- and LacZ-injected hearts, but tended to be higher in these groups than in non-injected hearts [27].

Pecam-1 (sc-1506-R, Santa Cruz Biotechnology, Santa Cruz, CA, USA) was used to stain endothelial cells. The number of capillaries was calculated from five representative high power fields (40×) from the LV of each section; 3 from epicardial and 2 from endocardial side of the LV were selected. To detect apoptotic cells, in situ labeling of the 3'-ends of the DNA fragments generated by apoptosis-associated endonucleases was performed using the ApopTag in situ apoptosis detection kit (Chemicon, Temicula, CA, USA), as previously described [28]. Briefly, DNA fragmentation was identified by applying terminal transferase enzyme with digoxigenin-labeled nucleotides. Anti-digoxigenin antibody was used to recognize the digoxigenin-labeled nucleotide chains attached to the 3'-ends of sample DNA. A color reaction was produced with diaminobenzidine and the sections were counterstained with hematoxylin. The apoptotic cells and bodies were counted in 5 high power fields (40× objective) choosing hot spot areas in each sample in order to make the results comparable. To determine whether TUNEL positive cells and proliferating cells were cardiomyocytes, cardiac fibroblasts or endothelial cells, immunofluorescence stainings were performed with alpha-actinin (ab28052, Abcam Inc., Cambridge, MA, USA), anti-prolyl 4-hydroxylase β (MAB2073, Millipore, Temecula,CA, USA) or Pecam-1 (sc-1506-R, Santa Cruz Biotechnology), respectively, and DAPI (diamidinophenylindole dihydrochloride, Sigma-Aldrich, St. Louis, MO, USA) as a nuclear stain.

To examine the efficiency and localization of the WDR12 gene delivery, the sections were incubated with specific polyclonal anti-WDR12 antibody (sc-132875, Santa Cruz Biotechnology, Santa Cruz, CA, USA) at the dilution of 1:50 3 days after gene transfer. Primary antibody for c-kit (sc-168, Santa Cruz Biotechnology, CA, USA) was used to stain stem cells. The number of c-kit+ cells in the anterior wall of LV was counted. The area of counted section was examined by computerized methods and a number of positively staining cells was related to the area (cells/35 mm^2^). To identify cells undergoing division, immunohistochemical labeling of nuclear Ki-67 or Phospho-histone-H3 antigen was performed by using monoclonal mouse anti-rat Ki-67 antigen antibody (DakoCytomation, Glostrup, Denmark) or Phospho-histone-H3 antigen antibody (Cell Signaling Technology, Beverly, MA, USA). The whole LV was scanned and stained cells were counted from high power fields (40×) choosing 5 hot spot areas in each sample. The primary antibodies were detected by peroxidase conjugated EnVision Detection Kit system (DakoCytomation, Denmark) and the samples were counterstained with haematoxylin. All measurements were performed blinded by persons, who were not aware of the treatments.

### Cell Culture

Neonatal rat ventricular myocytes were prepared from 2- to 4-day-old Spraque-Dawley rats as described earlier [29]. WDR12 or LacZ-adenoviruses were added to the cell culture medium 24h after the cells were plated and incubated 24h, at the virus concentration 6 MOI. The media were replaced every 24h, and 48h after infection, the cells were washed twice with PBS and quickly frozen at ‒70°C. For mechanical stretch experiments cardiomyocytes were plated on Bioflex plates and exposed to cyclic mechanical stretch as previously described [30]. Mechanical stretch was applied by Flexercell strain unit FX-5000. Adenoviral gene transfer of p38α and p38β as well as WDR12 in neonatal cardiomyocytes was performed as described previously [31].

Cardiac fibroblasts were separated from myocytes and plated on 10-cm dishes. On reaching confluences, fibroblasts were passaged into 6- or 12-well plates (170,000 or 70,000 cells/well, respectively). Specific siRNAs (WDR12 siRNA1 5’-GGGCCAGUUUCUUCGAAUGtt-3’ and WDR12 siRNA2 5’-GGAAAUAAGGUGUUCAAUUtt-3’) from Ambion, and negative control siRNA (mixed Ambion and Sigma-Aldrich) were transfected into the cardiomyocytes or cardiac fibroblasts using Lipofectamine 2000 (Invitrogen, Carlsbad, CA, USA) as transfection reagent. Cells were incubated in OPTI-mem (Invitrogen, Carlsbad, CA, USA) for 24h and thereafter cells were incubated in CSFM (myocytes) or DMEM+10% FBS (fibroblasts).

Total RNA was isolated from cell culture neonatal rat ventricular myocytes with TRIzol reagent (Invitrogen, Carlsbad, CA, USA) following the manufacturer’s protocol by using the Phase Lock Gel system (Eppendorf AG, Hamburg, Germany). Cultured cardiomyocytes were lysed in ice-cold lysis buffer (20 mM Tris-HCl, 150 mM NaCl, 1 mM EDTA, 1 mM EGTA, 1% Triton-X100, 2.5 mM sodium pyrophosphate, 1 mM β-glycerophosphate, 1 mM Na_3_VO_4_) supplemented with protease-inhibitor cocktail, phosphatase-inhibitor cocktail and 1mM DTT. The lysate was vortexed and then cleared by centrifugation at +4°C. The supernatant was then transferred in a new tube as the total protein extract.

The nuclear and cytosolic proteins were extracted adapting the protocol described by Schreiber *et al*. [32]. Cultured cardiomyocytes were washed and scraped with ice-cold 1x phosphate-buffered saline. After centrifugation the cell pellets were resuspended in 100 μl of low salt buffer consisting of 10 mM HEPES, 10 mM KCl, 0.1 mM EDTA and 0.1 mM EGTA supplemented with protease-inhibitor cocktail, phosphatase-inhibitor cocktail and 1 mM DTT. The suspensions were then allowed to swell on ice for 15 min. Then, membrane proteins were solubilised and isolated by adding 10 μl of 10% IGEPAL CA-630 detergent and vortexing vigorously followed by centrifugation. The supernatants were collected as the cytosolic fragments. The pellets were resuspended of high salt buffer containing 20 mM HEPES, 0.4 M NaCl, 1 mM EDTA and 1 mM EGTA, with supplements similar to those in low salt buffer and then rocked for 15 min. The samples were centrifuged and the supernatant was collected as the nuclear fragment. The entire procedure was carried out +4°C. Protein concentrations were determined with the Bio-Rad protein assay.

### Human LV Tissue Samples

LV samples were obtained from organ donors (*n* = 2) and patients with end-stage heart failure due to cardiomyopathy (*n* = 4) (2 patients were diagnosed with dilated cardiomyopathy, 1 with hypertrophic cardiomyopathy and 1 with idiopathic cardiomyopathy). The donor subjects had no cardiac history and no significant structural abnormalities on cardiac donor screening using echocardiography and coronary angiography. Donors were maintained under intensive care, but hearts were excluded ultimately from heart implantation because of age, size mismatch or blood type incompatibilities. After removal, cardiac tissue samples were blotted dry, immersed in liquid nitrogen, and stored at ‒80°C until assayed.

All patients were classified according to New York Heart Association (NYHA) functional classes III and IV. Transthoracic echocardiograms were performed using a 2.5-MHz linear transducer. Coronary angiographies were performed in all subjects using percutaneous femoral or radial approach by the Judkins technique. General patient characteristics are shown in Table 2.

**Table 2:** LV tissue samples were obtained from explanted human hearts in patients with cardiomyopathy undergoing heart transplantation.

Patient	A	B	C	D
Age (years)	44.9	50.9	47.2	53.8
Diagnosis	DCM	HCM	DCM	ICM
Gender	male	male	male	male
LVEF (%)	15	20	30	14
LA diameter (mm)	41	58	46	47
LVEDD (mm)	91	60	75	67
LVESD (mm)	83	51	62	62
sPA (mm Hg)	29	39	n.d.	30
RAP (mm Hg)	5	5	n.d.	15
E/E'	n.d.	10	n.d.	18

DCM, dilated cardiomyopathy; HCM, hypertrophic cardiomyopathy; ICM, ischemic cardiomyopathy; LVEF, left ventricular ejection fraction; LA, left atria; EDD, end diastolic diameter of the left ventricle; ESD, end systolic diameter of the left ventricle; sPA, systolic pulmonary artery pressure; RAP, right atrial pressure; n.d., not determined.

### Relationship between MI Associated WDR12 Allele and Cardiac Function

Malmö Preventive Project (MPP) started in the mid 1970s at the Malmö University Hospital, Malmö, Sweden as a prospective population-based study [33]. Between 1974 and 1992 a total of 33,346 men and women from the Malmö city area were recruited and screened for conventional risk factors for all-cause mortality and cardiovascular disease (CVD). Briefly, specially trained nurses performed the examinations in the morning. Participants were fasting overnight prior to investigation. Height and weight were measured in light indoor clothing without shoes. Blood samples were collected and biochemical parameters were analyzed by routine methods at the Department of Clinical Chemistry, Malmö University Hospital. Afterwards, participants answered a questionnaire consisting of approximately 260 questions using a self-administered computer program. The questions centered on personal and family history of CVD (myocardial infarction and stroke), hypertension, diabetes and cancer, as well as on conventional risk factors for these diseases. Antihypertensive treatment was defined as a positive answer to the following question: Do you take medication for high blood pressure? Blood pressure (BP) was measured in the supine position by specially trained nurses with a mercury sphygmomanometer and an appropriate cuff placed around the right arm at the level of the heart. In the years 2002–2006, all survivors from the original MPP cohort were invited to a follow-up examination. Of these, 18,240 participants responded to the invitation and were re-examined [34]. There were no exclusion criteria and of those who were alive the participation rate was 72%. For genetic studies, additional samples of peripheral venous blood were collected, as the original MPP design did not encompass DNA analyses.

In a sub-sample of the MPP follow-up examination, 1792 participants underwent echocardiography on a separate visit. As described in the original publication [35], subjects undergoing echocardiography were randomly selected from groups of glucometabolic status (normal fasting glucose, impaired fasting glucose, new onset diabetes mellitus and prevalent diabetes mellitus), with oversampling of the groups with glucometabolic disturbances). All subjects were studied in the left lateral decubital position. Conventional echocardiography/Doppler and tissue Doppler imaging (TDI) were conducted with a 3V2c transducer (Acuson Sequoia, Mountain View, CA, USA) or a S3 transducer (Sonos 5500 Philips, Andover, MA, USA). Parasternal long- and short-axis, and apical four- and two-chamber views were used to evaluate cardiac dimensions and LVEF [36]. Diastolic heart function was defined according to European Society of Cardiology guidelines [37] and we compared genotypic distribution of the previously myocardial infarction associated WDR12 SNP rs6725887 [5] between subjects who had normal diastolic function or only mild diastolic dysfunction (relaxation impairment) and those with significant diastolic dysfunction (i.e. subjects displaying a pseudonormalization pattern or a restrictive pattern). Left atrial area, indexed for body surface area, was used as a continuous trait for diastolic function. All analyses were performed offline without any knowledge of the subjects’ clinical status. Out of the 1792 subjects who underwent echocardiography, diastolic function could be determined in 1636 subjects and left atrial size in 1681 subjects. DNA was available and extracted from buffy coats with the use of QIAamp-96 spin blood kits (QIAGEN, Stockholm, Sweden) from 1496/1636 and 1531/1681 subjects and the rs6725887 SNP was successfully genotyped in 1394/1496 and 1436/1531 subjects using primers and probes which were custom synthesized by Applied Biosystems (Foster City, CA, USA) according to standard recommendations for the AB Prism 7900HT analysis system, and genotyped with polymerase chain reaction-based TaqMan method [38]. Detailed clinical characteristics of the 1436 subjects included in the genetic analyses of left atrial area are shown in Table 3.

**Table 3:** Clinical characteristics of the subjects included in the genetic analyses of left atrial area in Malmö Preventive Project (MPP).

Risk factor (n = 1436)	Average	min—max
Age (years)	67.7 ± 5.7	56.8–79.9
Gender (% male)	71.2	-
Systolic blood pressure (mmHg)	147 ± 19.8	94.5–253
Diastolic blood pressure (mmHg)	84.3 ± 4.2	56–135
Use of antihypertensive medication (%)	47.7	-
Body mass index (kg/m2)	28.2 ± 4.2	17.6–52.9
LDL-cholesterol (mmol/L)	3.5 ± 1.0	0.6–7.3
HDL-cholesterol (mmol/L)	1.3 ± 0.40	0.18–3.5
Current smokers (%)	18.3	-
Diabetes (%)	25.7	-

### Statistics

Results are expressed as mean±SEM. For statistical analyses, the data were first tested by using the Shapiro-Wilk test for normality. Because all variables were normally distributed, statistical significance was evaluated by 1-way ANOVA followed by a least significant difference (LSD) post hoc test for multiple comparisons. The Student *t* test was used for comparison between 2 groups. A probability value of <0.05 was considered statistically significant.

Crude and age- and sex-adjusted logistic regression models were used to relate rs6725887genotypes to a dichotomous dependent variable of diastolic dysfunction (presence of pseudonormalization pattern or restrictive pattern versus normal diastolic function or relaxation impairment). In crude and age- and sex-adjusted linear regression models we related rs6725887 genotypes to body surface normalized left atrial size and ejection fraction as a continuous variable.

## Results

### Cardiac Overload Induces WDR12 Expression through Direct Myocyte Stretch and p38 MAPK

**Fig 1:**
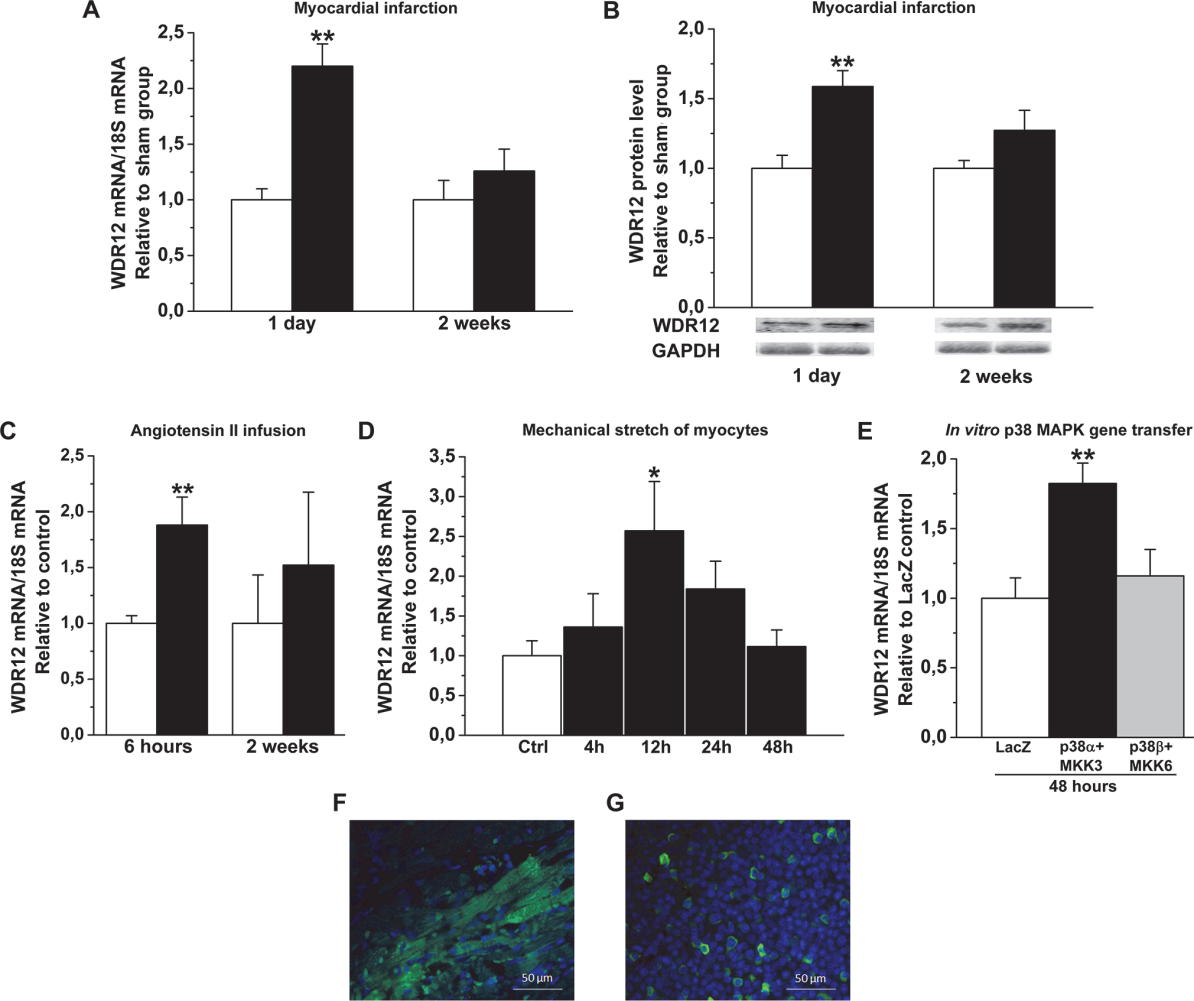
Induction of WDR12 expression by cardiac overload. **A.** Up-regulation of LV WDR12 mRNA, and **B**. protein levels by experimental MI at day 1 in rats. Representative Western blots are shown. Bands were detected from the same gel. Results are expressed as mean±SEM (n = 11 to 16). **P<0.01 MI (solid bars) versus sham (open bars) group (Student *t* test). **C**. Up-regulation of WDR12 mRNA levels in Ang II-induced hypertension at 6 hours. Results are expressed as mean±SEM (n = 6 to 8). **P<0.01 Ang II (solid bars) versus control (open bars) group (Student *t* test). **D.** Increase of WDR12 mRNA levels by mechanical stretch (n = 5 to 6), and **E**. p38 MAPK gene transfer (n = 6) *in vitro* in cell culture. The results are expressed as mean±SEM. *P<0.05, **P<0.01 versus control-group (1-way ANOVA followed by LSD post hoc test). **F**. Immunofluorescence staining of LV anterior wall, and **G**. thymus at day 1 post-infarction in rats. WDR12 is located in cardiac myocytes. WDR12 (green) and DAPI (blue).

Because WDR12 was associated with early-onset MI in GWAS studies [4,5], we first assessed the effect of post-infarction remodeling process on LV WDR12 gene expression. In response to MI induced by ligation of LAD in rats, WDR12 mRNA levels increased 2.2-fold (Fig 1A) and WDR12 protein levels 1.6-fold (Fig 1B) at day 1. When the effect of pressure overload on LV WDR12 expression was studied by infusing Ang II up to 2 weeks in rats, WDR12 mRNA levels were significantly elevated at 6 hours (1.9-fold)(Fig 1C). To characterize the potential mechanisms by which cardiac overload increases WDR12 expression, we employed an *in vitro* mechanical stretch model of cultured cardiomyocytes [39]. As shown in Fig 1D, direct mechanical stretch augmented WDR12 gene expression in neonatal rat cardiomyocytes, peaking at 12 h (2.6-fold). Moreover, once the role of the stretch-activated p38 MAPKs [25] in regulating WDR12 expression was studied, p38α but not p38β gene transfer *in vitro* increased WDR12 mRNA levels 1.8-fold (Fig 1E). To examine the LV localization of WDR12 we used immunofluorescence analysis with rat thymus [8] as a positive control. WDR12 was expressed in cardiac myocytes of rat hearts at 1 day post-infarction (Fig 1F and 1G).

### Augmentation of LV WDR12 Levels by Adenoviral Gene Delivery

**Fig 2:**
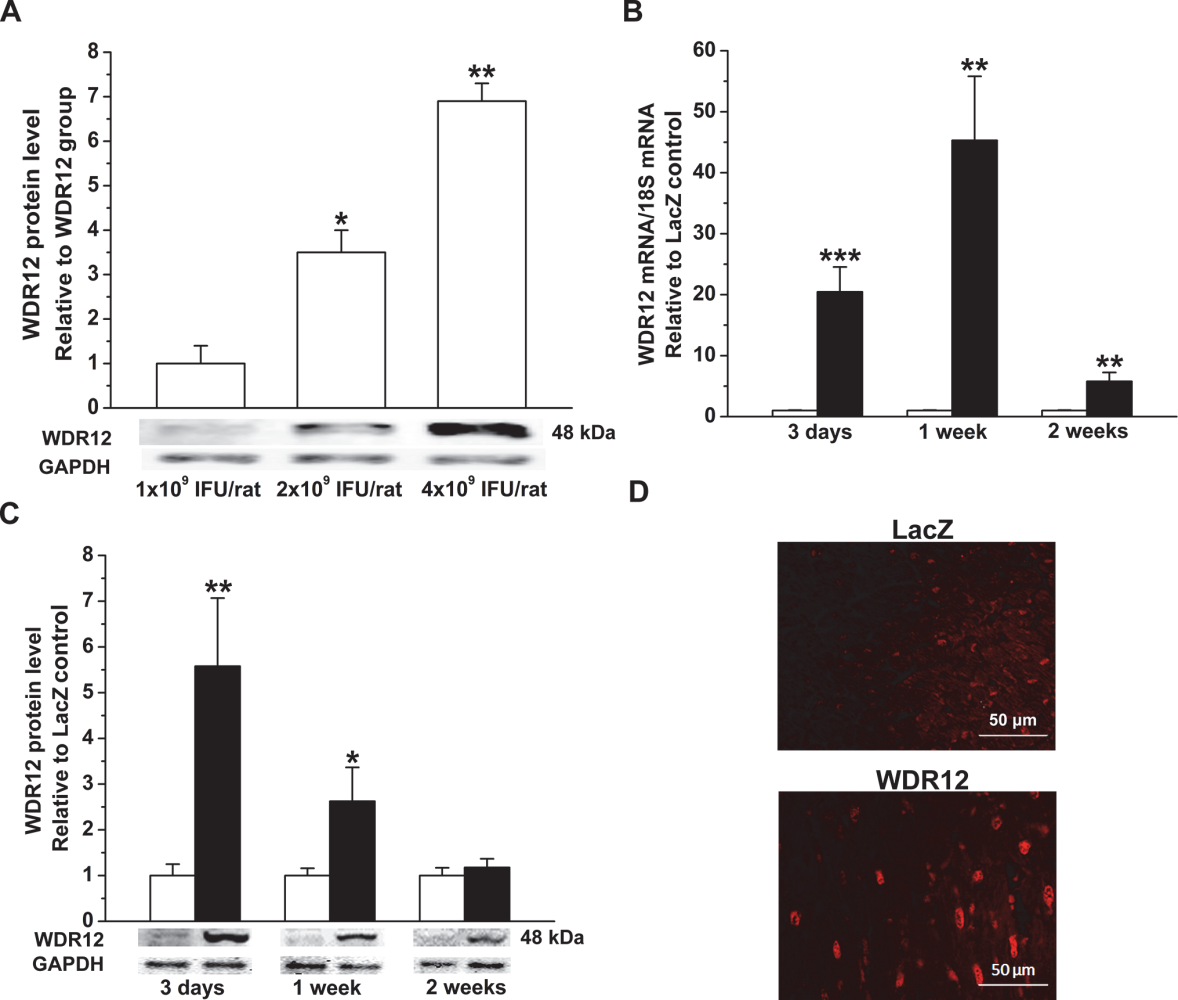
Cardiac specific activation of WDR12 by adenoviral gene delivery into the LV. **A.** Different doses of WDR12 adenoviral constructs were tested to increase LV WDR12 protein levels. Representative Western blots are shown. Bands were detected from the same gel. The results are expressed as mean±SEM (n = 3). *P<0.05, **P<0.01 versus 1x10^9^ IFU/rat injected group (1-way ANOVA followed by LSD post hoc test). **B.** WDR12 mRNA levels measured by RT-PCR, and **C.** WDR12 protein levels assessed by Western Blot analyses from the tissue samples 3 days, 1 week and 2 weeks after WDR12 gene delivery. Bands were detected from the same gel. Open bars represent LacZ and solid bars WDR12. The results are expressed as mean±SEM (n = 8 to 10). *P<0.05, **P<0.01, ***P<0.001 versus LacZ (Student *t* test). **D.** Immunofluorescence staining of LV anterior wall at day 3 after gene transfer showing WDR12 predominantly in cardiomyocytes (WDR12, red).

To study the direct myocardial effects of WDR12, we established an *in vivo* gene transfer protocol to locally increase LV WDR12 levels in the adult rat heart. As shown in Fig 2A, a dose-dependent increase in WDR12 protein levels, measured by western blot, was noted at day 3 after gene delivery. When WDR12 expressing adenoviral construct was injected into the LV free wall at 4x10^9^ ifu, WDR12 mRNA levels (Fig 2B) were highest at 1 week and protein levels (Fig 2C) at day 3 after injections and decreased thereafter to the levels of LacZ-treated hearts at 2 weeks. The increase in LV WDR12 protein levels at 1 week after gene delivery closely mimicked those increases observed in rats post-infarction and in response to pressure overload (see Fig 1B and 1C). The efficiency and localization of the WDR12 gene delivery was further confirmed by immunofluorescence. As shown in Fig 2D, immunofluorescence analysis showed augmented local staining in the cardiomyocyte nuclei of WDR12-treated hearts compared to LacZ-treated hearts.

### Local Myocardial WDR12 Gene Delivery Deteriorates Cardiac Function in Normal Adult Rats

**Fig 3:**
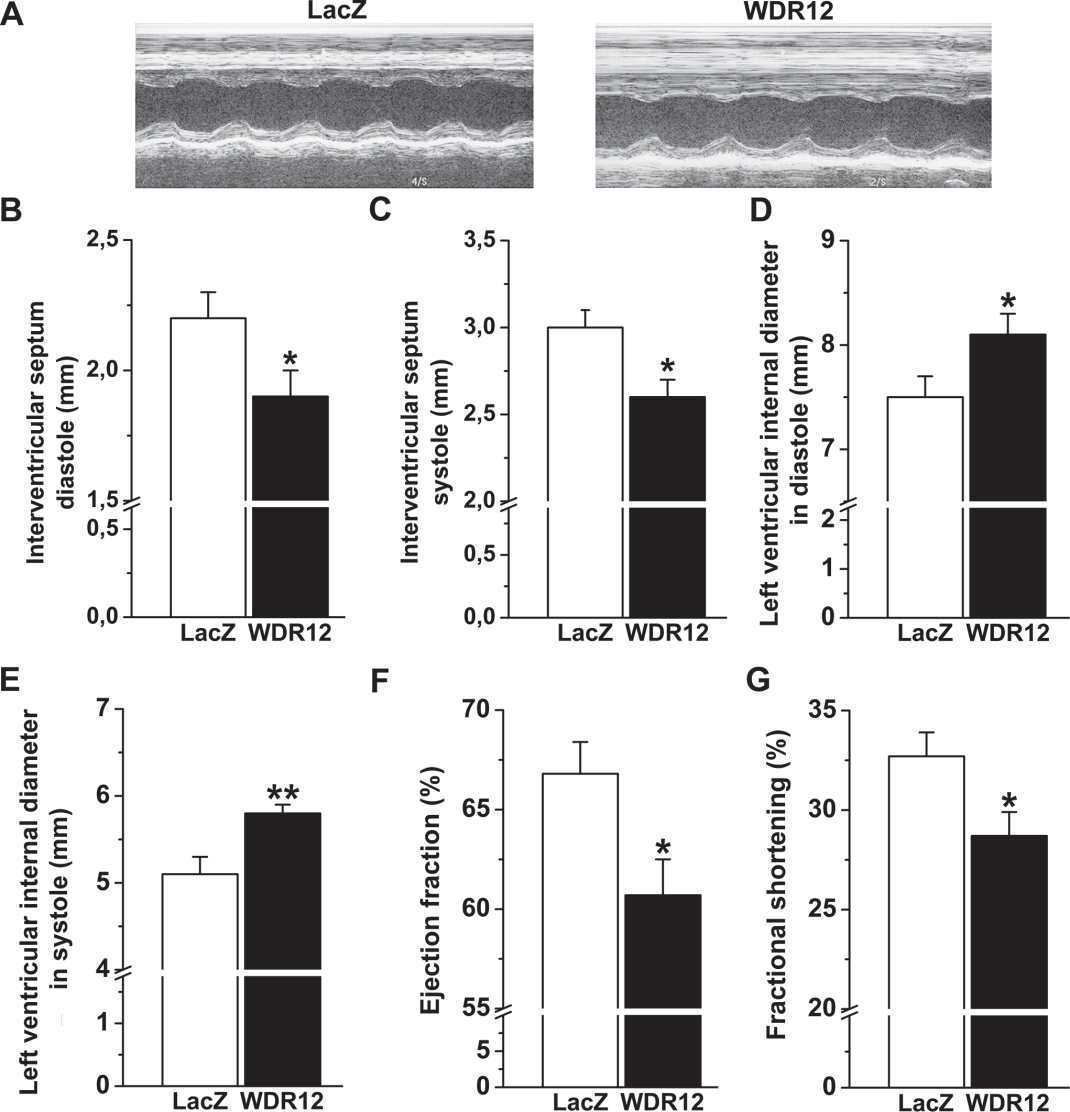
Intramyocardial WDR12 gene delivery deteriorates cardiac function in normal adult rat. Adenoviral construct expressing WDR12 and LacZ were injected into LV free wall. Echocardiographic measurements were performed 1 week after gene transfer. **A**. Representative M-mode images are shown. Treatment with WDR12 overexpressing vector significantly decreased interventricular septum diastolic (**B**) and systolic thickness (**C**), and increased LV diastolic (**D**) and systolic diameters (**E**). Overexpression of WDR12 decreased LV ejection fraction (**F**) and fractional shortening (**G**). The results are expressed as mean±SEM (n = 7 to 10). *P<0.05, **P<0.01 versus LacZ-control group (Student *t* test).

The effect of WDR12 gene delivery on cardiac function was analyzed by echocardiography (Fig 3A through 3G and Table 4) at 1 week following gene transfer. WDR12 gene transfer in normal rat hearts resulted in reduced interventricular septum thickness (Fig 3B and 3C) and increased LV diameters (Fig 3D and 3E) in both diastole and systole. Moreover, WDR12 gene delivery significantly decreased LVEF (*P*<0.05) and FS (*P*<0.05) (Fig 3F and 3G), collectively indicating that WDR12 gene delivery deteriorated cardiac function in normal adult rats.

**Table 4:** Effect of intramyocardial WDR12 gene delivery on cardiac structure and function in normal adult rat hearts, post-infarction, and in angiotensin II-induced hypertension at 1 week.

Variable	Group	Normal heart	MI	Ang II
**Interventricular septum thickness**				
diastole (mm)	LacZ	2.2 ± 0.1	0.9 ± 0.1	2.1 ± 0.1
	WDR12	1.9 ± 0.1*	1.2 ± 0.1**	2.2 ± 0.3
systole (mm)	LacZ	3.0 ± 0.1	1.0 ± 0.1	3.0 ± 0.1
	WDR12	2.6 ± 0.1*	1.3 ± 0.1	3.1 ± 0.4
**LV diameter**				
diastole (mm)	LacZ	7.5 ± 0.2	9.3 ± 0.2	7.3 ± 0.3
	WDR12	8.1 ± 0.2*	9.2 ± 0.2	7.2 ± 0.3
systole (mm)	LacZ	5.1 ± 0.2	8.1 ± 0.3	4.9 ± 0.3
	WDR12	5.8 ± 0.1**	8.1 ± 0.2	4.6 ± 0.3
**Posterior wall thickness**				
diatole (mm)	LacZ	1.7 ± 0.1	1.6 ± 0.1	2.0 ± 0.1
	WDR12	1.6 ± 0.1	1.6 ± 0.1	1.7 ± 0.2
systole (mm)	LacZ	2.6 ± 0.1	2.1 ± 0.1	2.8 ± 0.1
	WDR12	2.5 ± 0.1	2.2 ± 0.2	2.5 ± 0.1
**LV ejection fraction (%)**				
	LacZ	66.8 ± 1.6	31.9 ± 3.8	67.6 ± 3.9
	WDR12	60.7 ± 1.8*	29.0 ± 3.7	69.7 ± 3.2
**Fractional shortening (%)**				
	LacZ	32.7 ± 1.2	13.4 ± 1.8	34.0 ± 2.8
	WDR12	28.7 ± 1.2*	12.0 ± 1.7	35.5 ± 2.5
**IVRT**				
	LacZ	26.3 ± 1.0	27.4 ± 0.9	32.8 ± 1.2
	WDR12	27.4 ± 1.4	28.4 ± 0.8	35.3 ± 2.1
**E/A-ratio**				
	LacZ	5.0 ± 0.5	7.3 ± 0.8	2.5 ± 0.4
	WDR12	4.5 ± 0.4	5.0 ± 0.6*	1.8 ± 0.3

MI, myocardial infarction; Ang II, angiotensin II; LV, left ventricular; IVRT, isovolumic relaxation time

Adenoviral gene construct expressing WDR12 and LacZ were injected into LV free wall and echocardiographic measurements were performed at 1 week after gene transfer. The results are expressed as mean±SEM (n = 7–10).

*P<0.05,

**P<0.01 vs LacZ (Student *t* test).

### Effects of WDR12 Gene Delivery Post-Infarction and in Angiotensin II-Induced Hypertension

**Fig 4:**
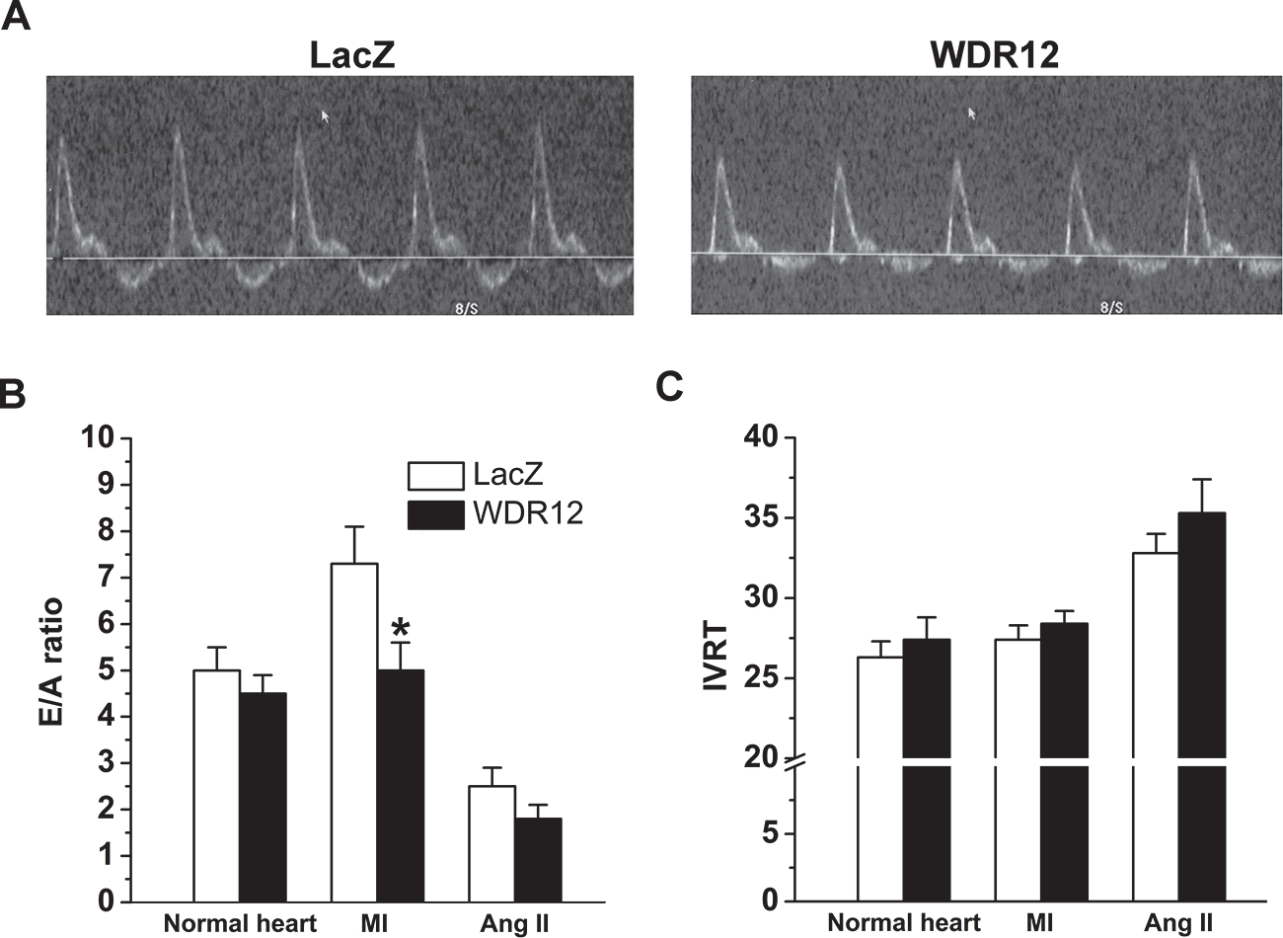
Intramyocardial WDR12 gene delivery decreases diastolic function in rats. Adenoviral construct expressing WDR12 and LacZ were injected into LV free wall. Echocardiographic measurements were performed 1 week after WDR12 gene transfer. **A**. Representative mitral flow velocity images of LacZ- and WDR12-injected infarcted hearts are shown. **B.** E/A ratio decreased significantly by intramyocardial WDR12 gene transfer in infarcted hearts, and non-significantly in WDR12-treated normal hearts and Ang II-infused hearts. **C**. IVRT values increased non-significantly by intramyocardial WDR12 gene transfer, in WDR12-treated normal hearts, infarcted hearts and Ang II-infused hearts. The results are expressed as mean±SEM (n = 7 to 10). *P<0.05 versus LacZ-control group (Student *t* test).

**Fig 5:**
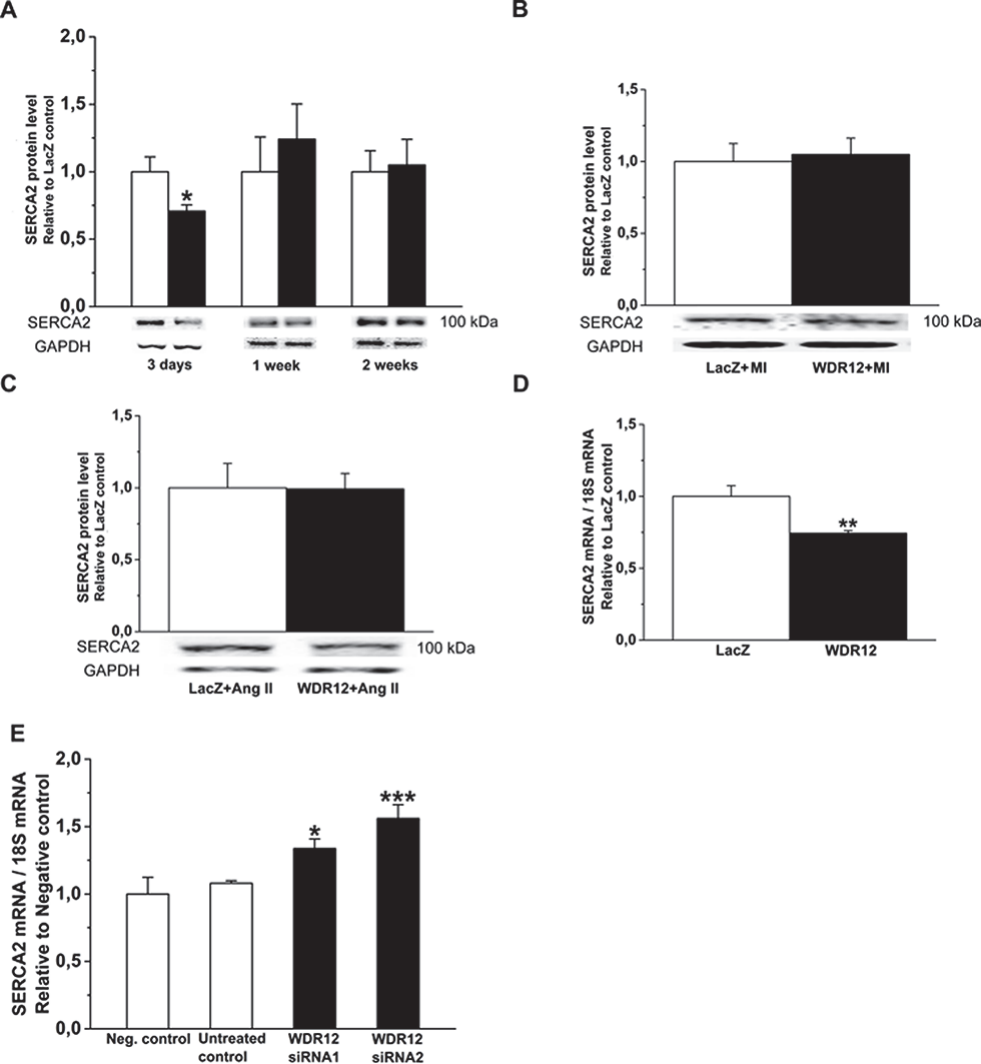
SERCA2 expression after intramyocardial WDR12 gene transfer and after WDR12 gene silencing. SERCA2 protein levels were assessed by Western Blot analyses from the LV samples 3 days, 1 week and 2 weeks after WDR12 gene delivery in normal adult rats (**A**), 1 week after myocardial infarction (**B**) and in angiotensin II (Ang II)-induced hypertension at 1 week (**C**) (n = 8 to 10). Bands were detected from the same gel. **D.** SERCA2 mRNA levels after adenoviral transfection of WDR12 *in vitro* in rat neonatal cardiomyocytes (n = 4). **A.** open bars represent LacZ and solid bars WDR12. *P<0.05, **P<0.01 versus LacZ (Student *t* test). **E.** SERCA2 mRNA levels after WDR12 siRNA transfection in rat neonatal cardiomyocytes (n = 4). *P<0.05, **P<0.001 versus negative control (1-way ANOVA followed by LSD post hoc test). The results are expressed as mean±SEM.

Because both MI and pressure overload caused significant up-regulation of WDR12 levels, we next examined the effects of WDR12 gene transfer on LV function and structure in MI induced by ligation of LAD and in angiotensin II-induced hypertension in rats. LV WDR12 mRNA and protein levels at 1 and 2 weeks after WDR12 gene transfer post-infarction and in Ang II-mediated hypertension increased similarly to those observed in normal adult rat hearts (Data not shown). As assessed by echocardiography, LV dimensions, LVEF and FS of WDR12-treated animals were similar to those of LacZ-treated hearts, while ligation of LAD alone caused a marked decrease in LVEF and FS (Table 4). Likewise, WDR12 gene transfer did not significantly affect LV dimensions, LVEF or FS in rats infused with Ang II (Table 4). However, evaluation of diastolic function revealed that WDR12 gene transfer decreased E/A ratio by 32% (*P*<0.05) at 1 week post-infarction (Fig 4A and 4B), suggesting that WDR12 gene delivery results in diastolic dysfunction. There was also a non-significant decrease in E/A ratio in normal rat hearts (13%) and in Ang II-mediated hypertension (27%)(Fig 4B). There was also a tendency towards increased isovolumic relaxation time (IVRT) at 1 week (Fig 4C). Interestingly, consistently with the impairment of cardiac function, LV SERCA2 protein levels were lower after WDR12 gene transfer at day 3 in normal adult rats, but remained unchanged in WDR12-treated hearts post-infarction and in Ang II-mediated hypertension (Fig 5A through 5C). Furthermore, SERCA2 mRNA levels decreased in response to adenoviral transfection of WDR12 *in vitro* in rat neonatal cardiomyocytes and increased by cell-specific silencing of WDR12 (Fig 5D and 5E).

### Marked Reduction of LV Cell Proliferation by WDR12 Overexpression

**Fig 6:**
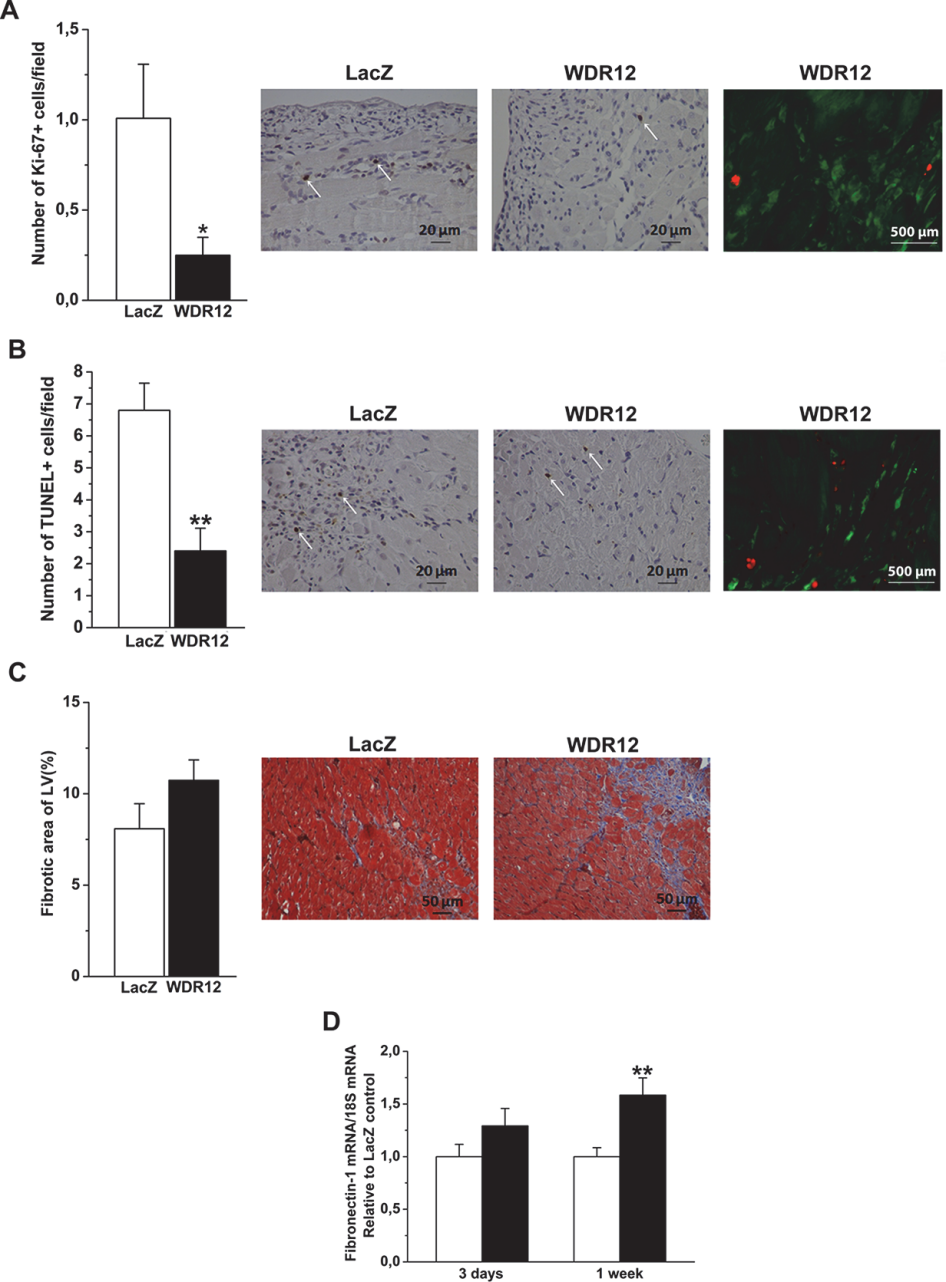
Myocardial cellular proliferation, apoptotic cell death and fibrosis in the LV after local WDR12 gene delivery at 1 week. **A**. Immunohistochemical staining against Ki-67 was performed to study the effect of WDR12 gene transfer on cellular proliferation. The whole left ventricle was scanned and stained cells were counted from high power fields (40x) choosing 5 hot spot areas in each sample. Immunofluorescence staining showing that proliferating cells were cardiac fibroblasts (red, Phospho-histone-H3; green, Prolyl 4-hydroxylase β). **B**. The rate of apoptosis was assessed by TUNEL. Immunofluorescence staining showing that apoptotic cells were cardiac fibroblasts (Red, TUNEL; green, Prolyl 4-hydroxylase β). **C.** Masson’s trichrome staining was used to define area of fibrosis by computerized methods. The results are expressed as mean±SEM (n = 4 to 5). **D**. Fibronectin-1 mRNA levels were increased 1 week after WDR12 gene delivery. Open bars represent LacZ and solid bars WDR12. The results are expressed as mean±SEM (n = 8 to 10). *P<0.05, **P<0.01 versus LacZ (Student *t* test).

**Fig 7:**
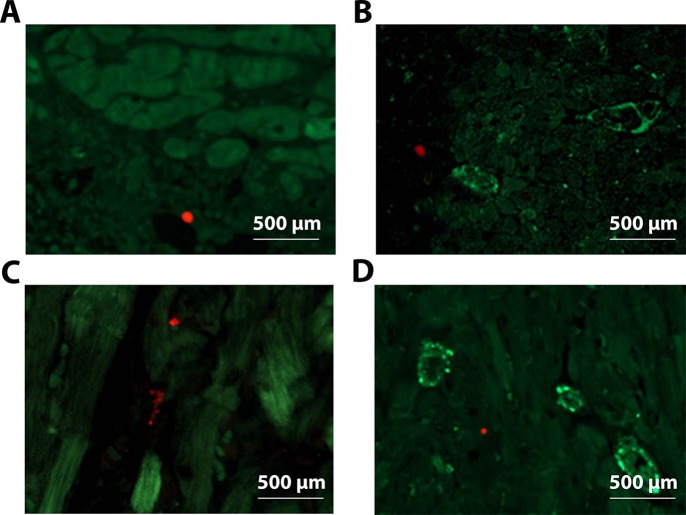
Immunofluorescence staining showing that proliferating cells and apoptotic cells were non-cardiomyocytes and non-endothelial cells. **A.** Proliferating cells (red, Phospho-histone-H3; green, α-actinin). **B.** Proliferating cells (red, Phospho-histone-H3; green, Pecam-1). **C.** Apoptotic cells (red, TUNEL, green, α-actinin), **D.** Apoptotic cells (red, TUNEL, green, Pecam-1).

**Fig 8:**
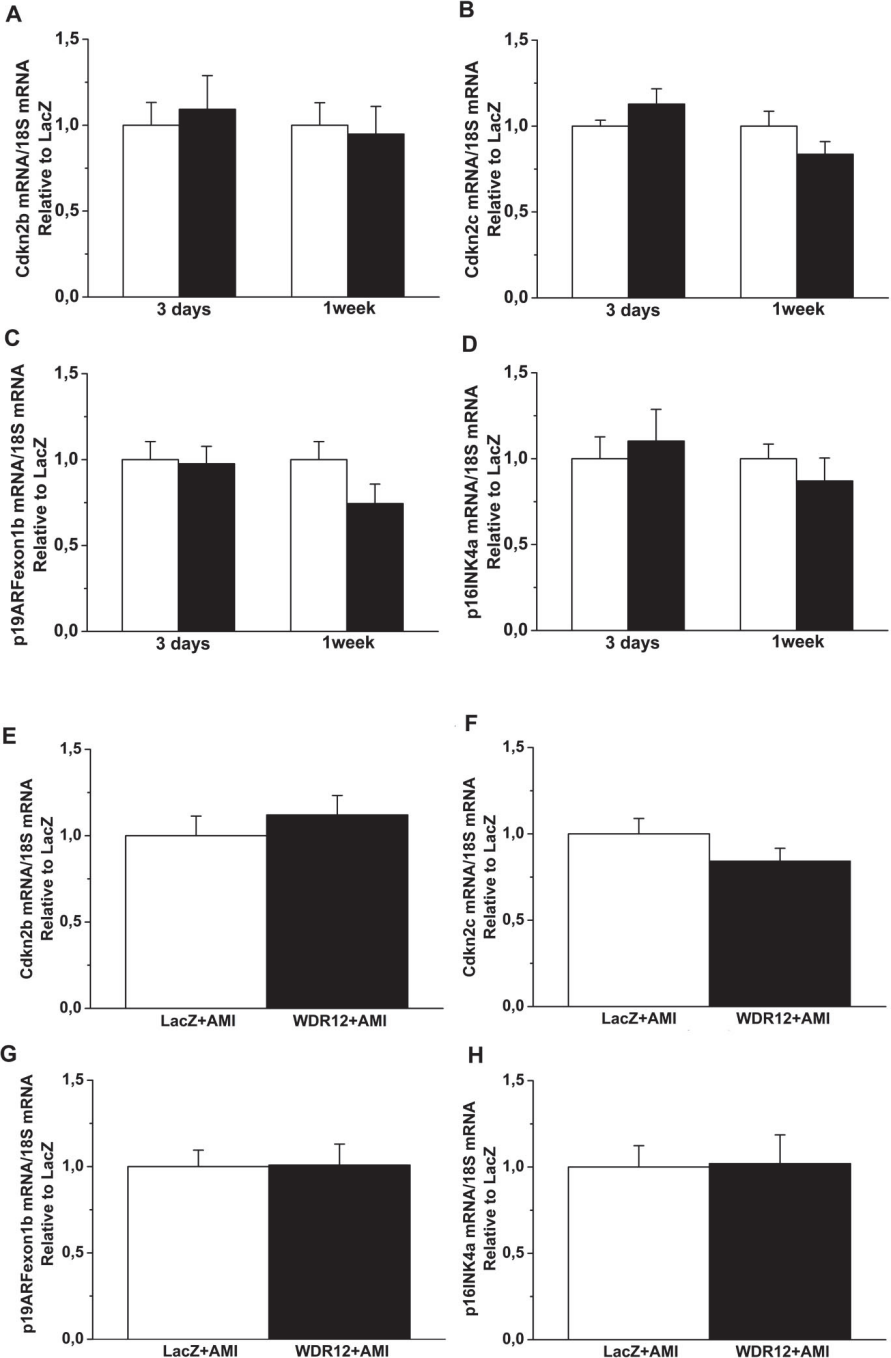
Effects of WDR12 gene delivery on the expression of putative WDR12 target genes in normal rat heart and during 1 week post-infarction. **A**. Cyclin-dependent kinase inhibitor 2 b (Cdkn2b), **B**. Cdkn2c, **C**. p19ARFexon1b, **D**. p16INK4a mRNA levels in normal rat heart and **E**. Cdkn2b, **F**. Cdkn2c, **G**. p19ARFexon1b, **H**. p16INK4a mRNA levels at 1 week post infarction. **A-D**. Open bars represent LacZ and solid bars WDR12. The results are expressed as mean±SEM (n = 7–10). P = ns versus LacZ (Student *t* test).

Since PeBoW complex regulates cell proliferation [9], we next assessed whether the changes in cardiac structure and function after WDR12 gene delivery were related to the alterations in cell proliferation by staining histological sections immuno-histochemically against nuclear Ki-67. As shown in Fig 6A, intramyocardial WDR12 gene delivery resulted in a marked decrease (75%) in the number of Ki-67 positive cells at 1 week. Double immunofluorescence staining of proliferating cells showed that they were positive for fibroblast marker prolyl 4-hydroxylase (Fig 6A) but not for cardiomyocyte or endothelial cell markers (Fig 7A and 7B). We also analyzed histological sections for apoptotic cell death, myocardial fibrosis, cardiac stem cell recruitment and angiogenesis. As assessed by TUNEL assay, WDR12 gene transfer significantly decreased apoptotic cell death (65%)(Fig 6B). Double immunofluorescence staining of TUNEL+ cells showed that also they were positive for fibroblast marker prolyl 4-hydroxylase (Fig 6B) but not for cardiomyocyte or endothelial cell markers (Fig 7C and 7D). Moreover, there was a non-significant increase in myocardial fibrosis, measured by staining histological sections with Masson’s trichrome (Fig 6C), and a significant increase in LV expression of a fibrosis-related gene, fibronectin-1 (Fig 6D). On the other hand, the number of c-kit+ cells or coronary angiogenesis did not significantly differ between LacZ- and WDR12-treated hearts (Data not shown). Finally, because dominant-negative mutant of WDR12 has been shown to block rRNA processing, induce a reversible cell cycle arrest, and trigger accumulation of p53 in a p19ARF-independent manner in proliferating cells [9], we examined the effects of WDR12 gene delivery on the LV mRNA levels of selected regulators of the cell cycle. However, no significant differences in cyclin-dependent kinase inhibitor 2b and 2c, p19ARF and p16INK4a mRNA levels were observed between WDR12-treated and LacZ-treated normal rat hearts and post-infarction at 1 week after gene transfer (Fig 8).

### Regulation of BOP1 and Pes Protein Levels by WDR12

**Fig 9:**
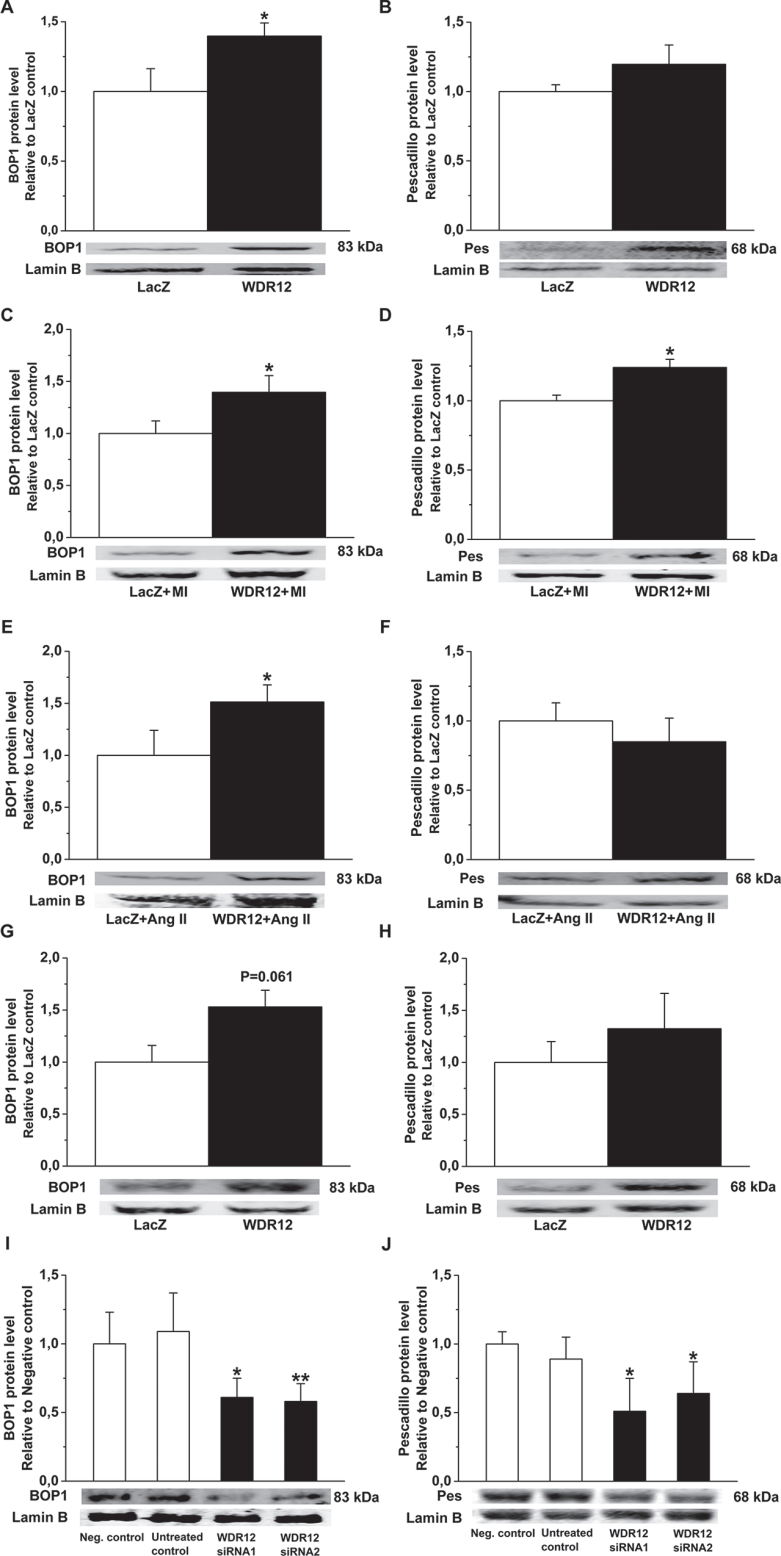
Effects of intramyocardial WDR12 gene delivery and WDR12 gene silencing on BOP1 and Pes nuclear protein expressions. **A**. BOP1, and **B**. Pes protein expression increased in normal rat hearts 1 week after gene transfer. **C**. BOP1, and **D**. Pes proteins were up-regulated by WDR12 gene transfer in infarcted hearts. **E**. BOP1 protein expression increased in Ang II-induced hypertension, but no differences in Pes protein level was noted **(F)**. **G.** and **H.** BOP1 and Pes protein levels after adenoviral transfection of WDR12 *in vitro* in rat neonatal cardiomyocytes. The results are mean±SEM (n = 7 to 10). *P<0.05 versus LacZ-control group (Student *t* test). **I.** and **J**. BOP1 and Pes protein levels after WDR12 siRNA transfection in rat neonatal cardiomyocytes (n = 4). *P<0.05, **P<0.01 versus negative control (1-way ANOVA followed by LSD post hoc test). Representative Western blots are shown. Bands were detected from the same gel.

**Fig 10:**
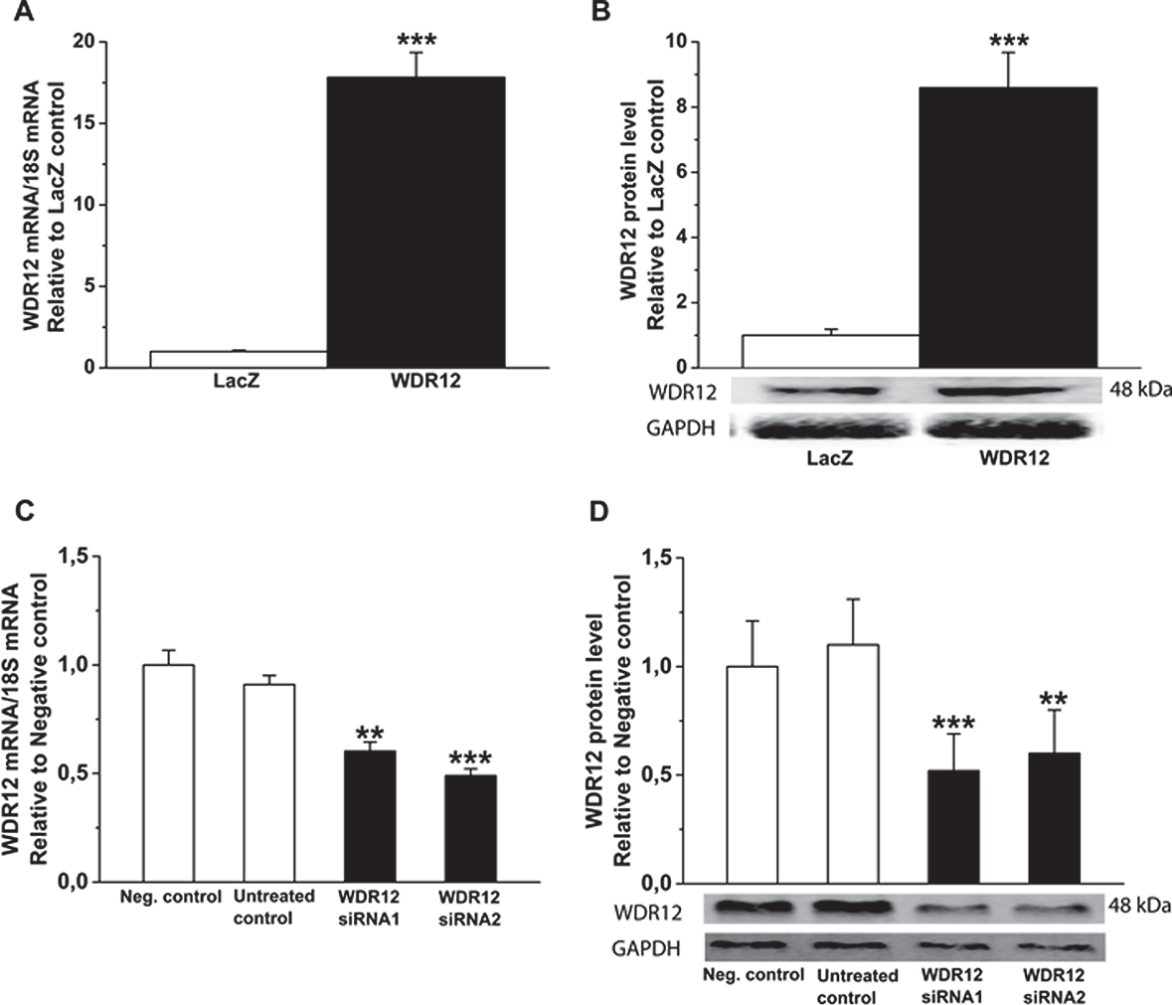
Activation and silencing of WDR12 in rat neonatal cardiac myocytes *in vitro*. **A.** WDR12 mRNA levels measured by RT-PCR, and **B.** WDR12 protein levels assessed by Western Blot analyses from total protein cell lysates 48 hours after WDR12 gene transfer (n = 8). ***P<0.001 versus LacZ-group (Student *t* test). **C.** WDR12 mRNA and **D**. WDR12 protein levels after WDR12 siRNA transfection (n = 4). **P<0.01, ***P<0.001 versus negative control group (1-way ANOVA followed by LSD post hoc test). The results are expressed as mean±SEM. Representative Western blots are shown. Bands were detected from the same gel.

**Fig 11:**
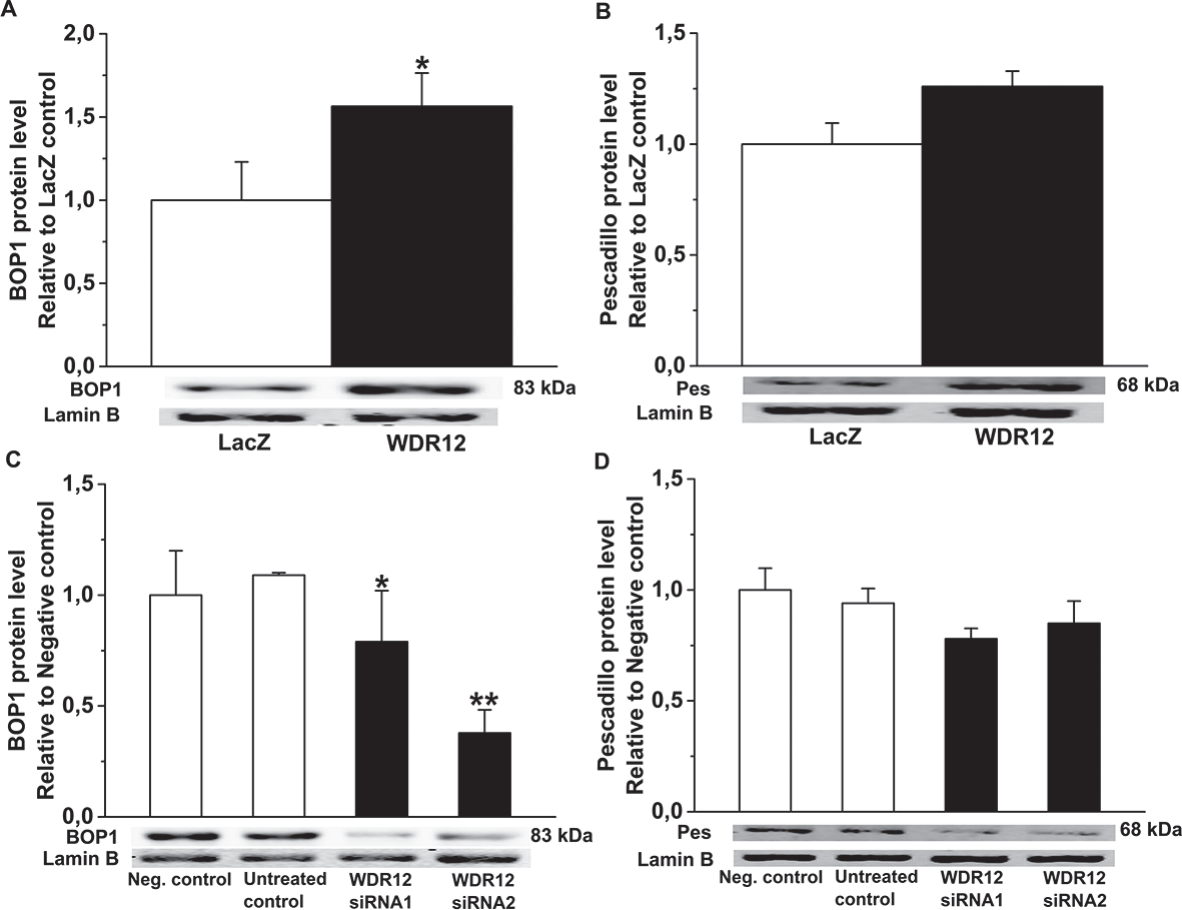
Effects of WDR12 gene delivery and WDR12 gene silencing *in vitro* in rat neonatal fibroblasts. **A.** BOP1 and **B.** Pes protein levels after adenoviral transfection of WDR12. *P<0.05 versus LacZ-control group. **C.** BOP1 and **D.** Pes protein levels after WDR12 siRNA transfection *P<0.05, **P<0.01 versus negative control group (1-way ANOVA followed by LSD post hoc test). The results are mean±SEM (n = 4). Representative Western blots are shown. Bands were detected from the same gel.

**Fig 12:**
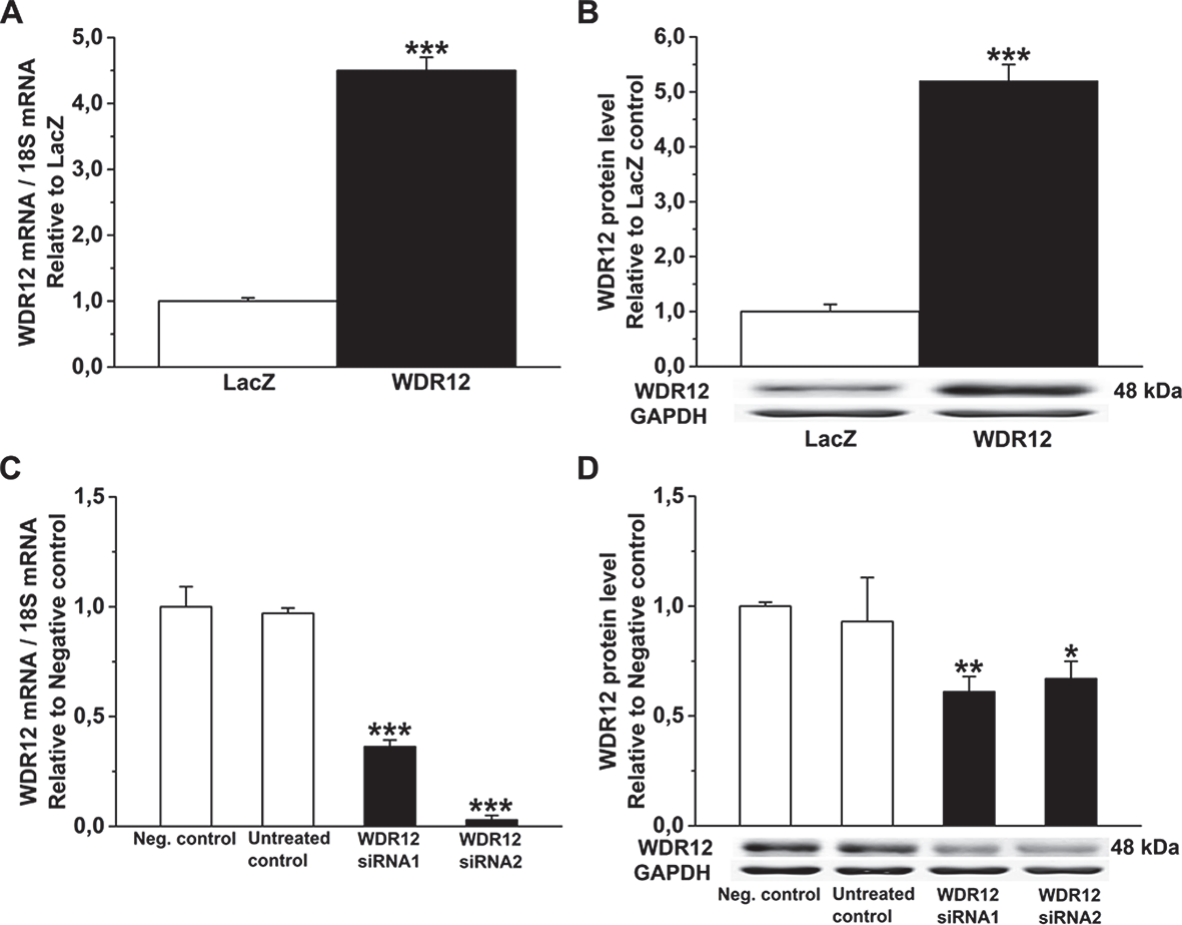
Activation and silencing of WDR12 in rat neonatal cardiac fibroblasts *in vitro*. **A.** WDR12 mRNA levels measured by RT-PCR, and **B.** WDR12 protein levels assessed by Western Blot analyses from total protein cell lysates after WDR12 gene transfer. ***P<0.001 versus LacZ-group (Student *t* test). **C.** WDR12 mRNA and **D.** WDR12 protein levels after WDR12 siRNA transfection (n = 4). *P<0.05, **P<0.01, ***P<0.001 versus negative control group (1-way ANOVA followed by LSD post hoc test). The results are expressed as mean±SEM (n = 4). Representative Western blots are shown. Bands were detected from the same gel.

Because WDR12 forms a stable complex and directly interacts with nucleolar target genes Pes1 and BOP1 in rRNA processing and proliferation [40,41], we next examined protein levels of BOP1 and Pes after WDR12 gene transfer by Western blot analysis. Remarkably, WDR12 overexpression consistently increased BOP1 in normal adult rat hearts (*P*<0.05)(Fig 9A), post-infarction (*P*<0.05)(Fig 9C), in Ang II-induced hypertension (*P*<0.05)(Fig 9E) and *in vitro* in neonatal rat cardiomyocytes (p = 0.061)(Fig 9G and Fig 10A and 10B) and in cardiac fibroblasts (Fig 11A and Fig 12A and 12B). Pes protein levels increased significantly by WDR12 gene transfer only at 1 week post-infarction (Fig 9B, 9D, 9F and 9H and Fig 11B). Moreover, BOP1 and Pes protein levels were decreased after WDR12 siRNA transfection in both neonatal cardiomyocytes (Fig 9I and 9J and Fig 10C and 10D) and fibroblasts (Fig 11C and 11D and Fig 12C and 12D).

### WDR12 activates p38 MAPK/HSP27 and ERK1/2 Pathways

**Fig 13:**
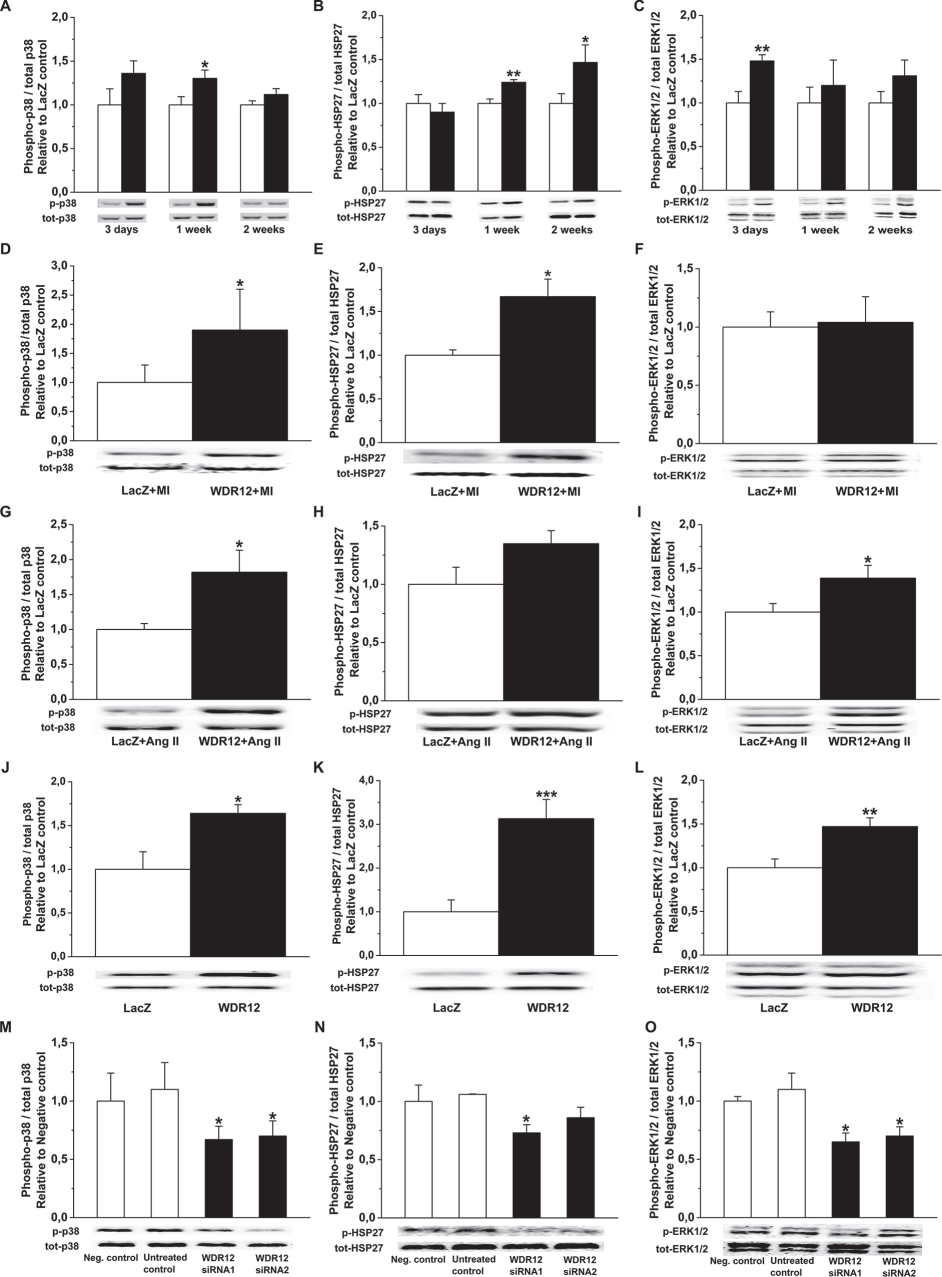
Regulation of p38 MAPK, HSP27 and ERK1/2 phosphorylations by WDR12. **A.** and **B.** p38 MAPK and HSP27 phosphorylations were up-regulated in normal heart, **D.** and **E.** after infarction, and **G.** and **H.** p38 MAPK in Ang II-induced hypertension at 1 week after WDR12 gene delivery. **C.** ERK1/2 phosphorylations were up-regulated in normal heart at 3 days, and **I**. in Ang II-induced hypertension at 1 week, but not post-infarction **(F)**. **J. K.** and **L.** p38 MAPK, HSP27 and ERK1/2 phosphorylations were increased by adenoviral transfection of WDR12 *in vitro* in rat neonatal cardiomyocytes (n = 7 to 10). **A-C.** open bars represent LacZ and solid bars WDR12. *P<0.05, **P<0.01, ***P<0.001 versus LacZ-control group (Student *t* test). **M. N.** and **O.** p38 MAPK, HSP27 and ERK1/2 phosphorylations were decreased by WDR12 siRNA transfection *in vitro* in rat neonatal cardiomyocytes (n = 4). *P<0.05 versus negative control group (1-way ANOVA followed by LSD post hoc test). The results are mean±SEM. Representative Western blots are shown. Bands were detected from the same gel.

**Fig 14:**
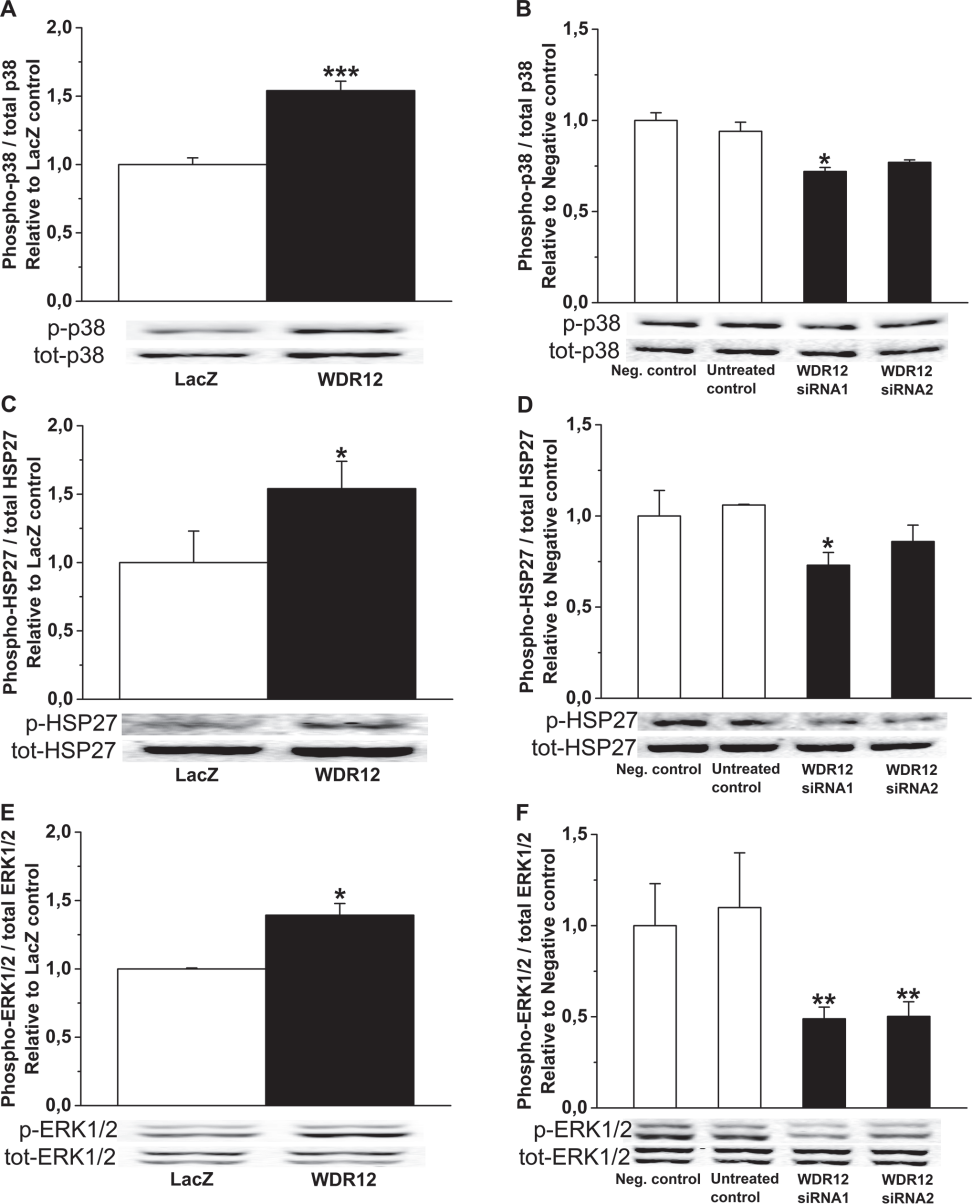
Regulation of p38 MAPK, HSP27 and ERK1/2 phosphorylation by WDR12 *in vitro* in rat neonatal cardiac fibroblasts. **A. C.** and **E.** p38 MAPK, HSP27 and ERK1/2 phosphorylations were increased by adenoviral transfection of WDR12. *P<0.05, ***P<0.001 versus LacZ-control group (Student *t* test). **B. D.** and **F.** p38 MAPK, HSP27 and ERK1/2 phosphorylations were decreased by WDR12 siRNA transfection *in vitro* in rat neonatal cardiac fibroblasts. *P<0.05, **P<0.01 versus negative control group (1-way ANOVA followed by LSD post hoc test). The results are mean±SEM (n = 4). Representative Western blots are shown. Bands were detected from the same gel.

To further evaluate potential mechanisms triggering structural changes and LV dysfunction by local WDR12 gene delivery, we assessed changes in the phosphorylations of p38 MAPK, ERK1/2 and HSP27, involved in regulation of cell proliferation, differentiation and survival [42,43]. WDR12 gene delivery significantly increased both p38 MAPK/HSP27 and ERK1/2 phosphorylations in the adult rat hearts at 1 week (Fig 13A through 13C). Up-regulation of WDR12 levels produced increase of p38 MAPK and HSP27 but not ERK1/2 phosphorylation also post-infarction (Fig 13D through 13F). In Ang II-induced hypertension, WDR12 gene delivery significantly increased both p38 MAPK and ERK1/2 phosphorylations and non-significantly HSP27 phosphorylation (Fig 13G through 13I). Finally, p38 MAPK, HSP27 and ERK1/2 phosphorylations were up-regulated after adenoviral transfection of WDR12 *in vitro* in rat neonatal cardiomyocytes (Fig 13J through 13L) and fibroblasts (Fig 14A, 14C and 14E) associated with a 17.8-fold increase in WDR12 mRNA and 8.6-fold increase in WDR12 protein levels in myocytes (Fig 10A and 10B) and 4.5-fold increase in WDR12 mRNA and 5.2-fold increase in WDR12 protein levels in fibroblasts (Fig 12A and 12B). Furthermore, WDR12 siRNA gene silencing *in vitro* resulted in decreased phosphorylation of p38 MAPK, HSP27 and ERK1/2 in neonatal myocytes (Fig 13M, 13N and 13O and Fig 10C and 10D) and in fibroblasts (Fig 14B, 14D and 14F and Fig 12C and 12D).

### Augmented LV WDR12 Expression in Patients with cardiomyopathy

**Fig 15:**
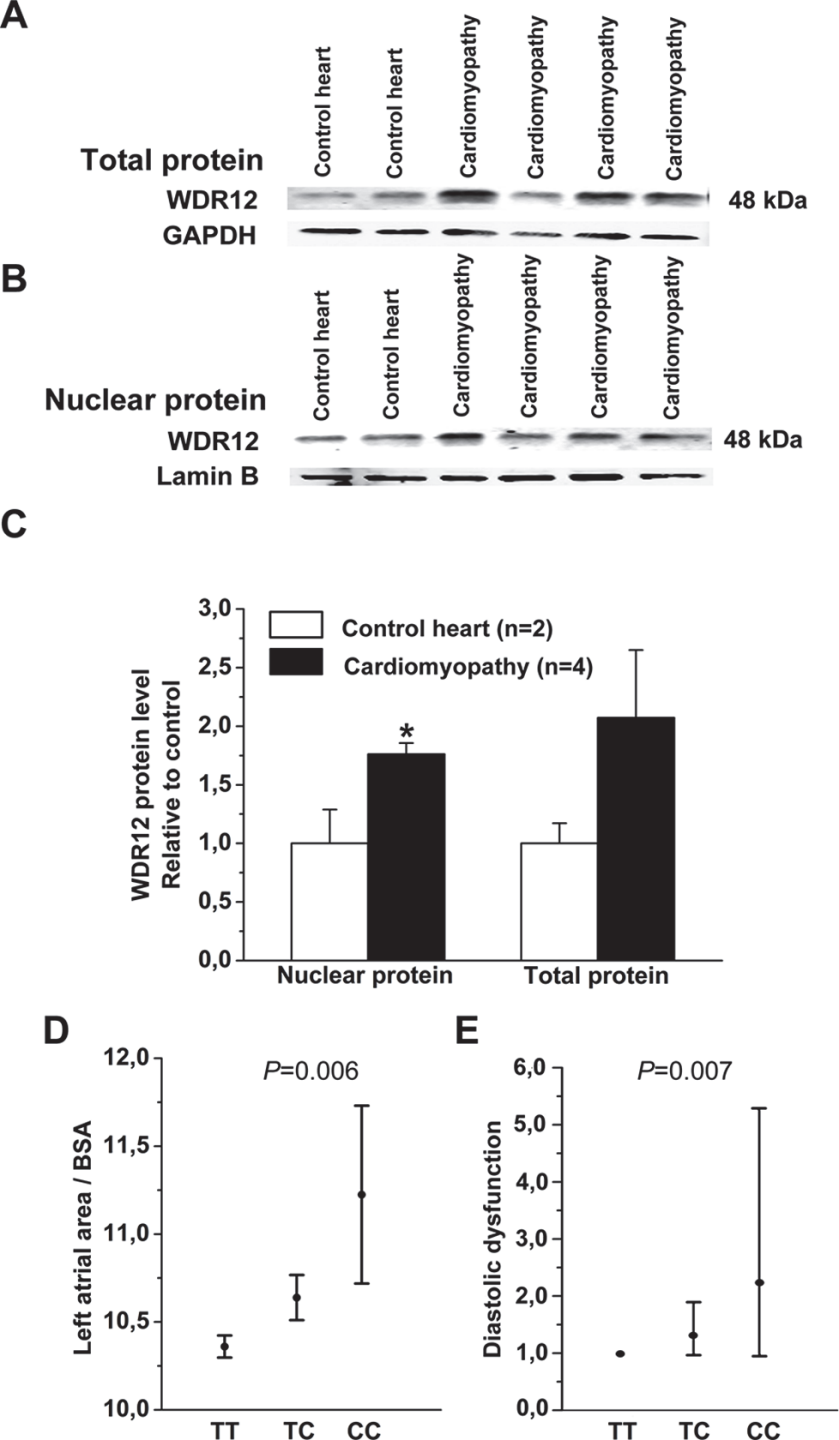
WDR12 protein levels in human LVs and association of MI associated variant with echocardiographic parameters. Representative Western blots are shown. **A**. WDR12 total protein and **B**. WDR12 nuclear protein levels in patients with cardiomyopathy and donor control hearts. Bands were detected from the same gel. **C**. WDR12 protein levels relative to donor control hearts. The results are expressed as mean±SEM. *P<0.05 versus control group (Student *t* test). **D**. Left atrial area/BSA (cm^2^/m^2^) in carriers of the previously myocardial infarction associated rs6725887 WDR12 genotypes and **E**. odds ratio (95% confidence intervals) for diastolic dysfunction (pseudonormalization + restrictive versus normal + relaxation impairment) in carriers of the previously myocardial infarction associated rs6725887 WDR12 genotypes.

WDR12 protein was also found to be expressed in the LV of control human hearts and in patients with cardiomyopathy (Fig 15A and 15B). Nuclear WDR12 protein levels were higher (1.8-fold) in patients with cardiomyopathy than in control hearts (Fig 15B and 15C), and there was also a non-significant increase (2.1-fold) in total WDR12 protein levels in patients with cardiomyopathy when compared to control hearts (Fig 15A and 15C). Also WDR12 mRNA as well as ANP and BNP mRNA levels were higher in patients with cardiomyopathy (Data not shown).

### MI Associated WDR12 Allele Associates with Diastolic Dysfunction in Humans

In a crude additive model, the MI associated allele of rs6725887 (minor allele) was significantly associated with larger left atrial area/BSA (β-coefficient±SEM)(0.32±0.12, *P* = 0.006), which remained significant after age and sex adjustment (0.28±0.12, *P* = 0.016)(Fig 15D). Presence of significant diastolic dysfunction (pseudonormalization or restrictive pattern) was significantly associated with the MI associated allele of rs6725887 in crude additive model (odds ratio, 95% confidence interval) (1.42, 1.10‒1.83, *P* = 0.007) as well as after age and sex adjustment (1.38, 1.07‒1.79, *P* = 0.013)(Fig 15E). The association remained significant after additional adjustment for history of myocardial infarction (1.41, 1.09–1.82, *P* = 0.009). In contrast, there was no significant association between rs6725887 and variation of systolic function (LVEF) (*P* = 0.616).

## Discussion

While recent genome-wide association studies have associated WDR12 with early-onset MI and CAD in humans [4,5], nothing is known of the function of WDR12 in the heart. In the present study we found that LV WDR12 expression was elevated in patients with cardiomyopathy, in rats post-infarction and in Ang II-mediated hypertension. By using local intramyocardial adenovirus-mediated gene delivery, we provide *in vivo* evidence that WDR12 overexpression deteriorated LV systolic and diastolic function in adult rat heart. Moreover, MI associated allele of WDR12 was associated with diastolic dysfunction in humans. Collectively, these results demonstrate that WDR12 induces worsening of LV function and suggest WDR12 as a potential novel therapeutic target for treatment of failing hearts.

Cellular processes often depend on stable physical associations between proteins. WDR12, a member of nucleolar PeBoW-complex, has been implicated in variety cellular processes such as pre-RNA processing, signal transduction, cell division, cell cycle progression, apoptosis and gene regulation [8–10]. A previous study has suggested that the BOP1/WDR12 subcomplex is retained in the cytoplasm, whereas BOP1/Pes1 is located in the nucleolus [10]. In addition, it has been reported that cytoplasmic Pes1, BOP1 and WDR12 are unstable and that BOP1 requires Pes1 for nucleolar transport [10]. Yet, the same researchers have found that overexpressed WDR12 is mainly located in the nucleolus [9], suggesting that nucleolar transport of WDR12 also occurs independently of Pes1 and BOP1 proteins and that PeBoW complex assembles in the nucleolus. In our present study, nuclear WDR12 protein levels were elevated in LV of patients with cardiomyopathy, and there was also a non-significant increase in total WDR12 protein levels. Moreover, while WDR12 overexpression *in vivo* resulted in local staining in the cardiomyocyte nuclei, high levels of WDR12 cytoplasmic protein were also observed demonstrating the presence of both nucleolar and cytoplasmic WDR12 expression, and that, at least partially, cytoplasmic WDR12 is also stable in myocytes. Of note, the transient expression of WDR12 protein might be a consequence of post-translational modification of overexpressed protein or neutralizing antibodies. Indeed, several previous studies have reported that the immunogenicity of transgene proteins is a primary determinant of the temporal transgene expression [44–46].

**Fig 16:**
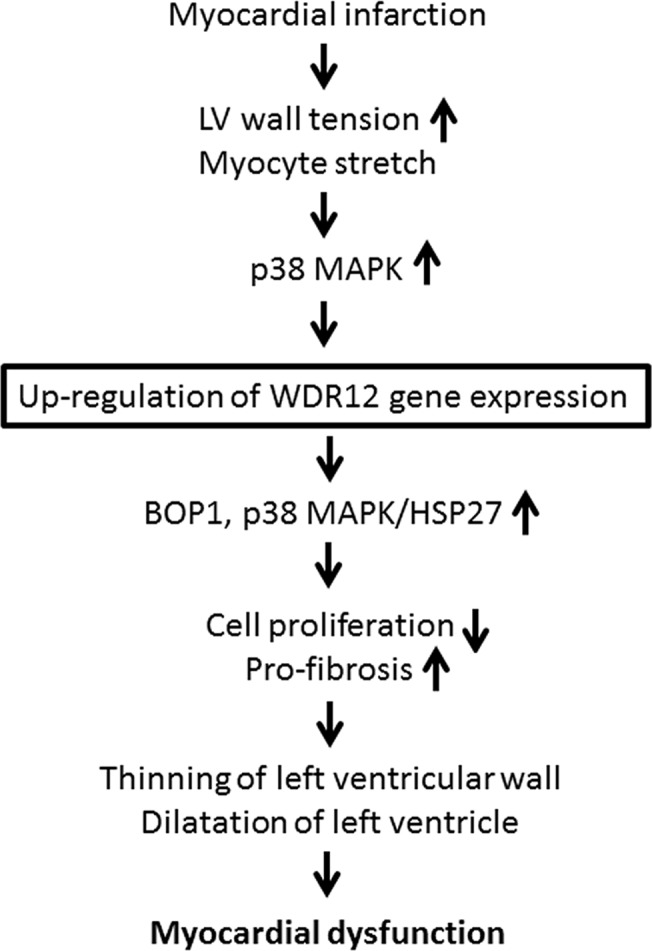
A schematic presentation of WDR12 in the heart. Our results suggest that in MI increased LV wall tension induces up-regulation of WDR12 expression via direct myocyte stretch and activation of p38 MAPK pathway. WDR12 gene delivery activated BOP1 and p38 MAPK/HSP27 pathways, leading to decreased cell proliferation and pro-fibrosis, thinning of the LV wall and dilatation of the LV, and myocardial dysfunction.

The present study shows that LV WDR12 mRNA and protein levels increase rapidly in response to cardiac overload (within 6–24 hours) suggesting that WDR12 may have a role in an early-phase remodeling process post-infarction and in pressure overload in rats. Further characterization of the role of cardiomyocytes in cardiac overload-induced WDR12 gene expression revealed that stretching of cultured neonatal myocytes resulted in up-regulation of WDR12 mRNA levels. In cardiac myocytes, mechanical stretch induces the MAPK cascade, resulting in the activation of three terminal MAP kinases, p38 kinase, ERK and c-jun N-terminal protein kinase [25,42]. Since p38α but not p38β gene transfer *in vitro* increased WDR12 mRNA levels, our results suggest that cardiac overload induces WDR12 expression in the heart via p38α MAPK activation induced by direct mechanical myocyte stretch (Fig 16). Of note, both post-infarction remodeling and Ang II-induced hypertension involve early cardiac activation of p38 MAPK [42].

A key finding of our present study was that in normal adult rat hearts WDR12 gene delivery into the anterior wall of the LV decreased interventricular septum diastolic and systolic thickness and increased diastolic and systolic diameters of the LV. Moreover, LVEF and FS were reduced indicating deteriorated systolic function. It is noteworthy that changes in LVEF and FS as well diameters of the LV and septum thickness by WDR gene delivery were similar in magnitude than those produced by the 2 weeks angiotensin II infusion [27], indicating that the changes in cardiac function and structure were physiologically relevant. However, WDR12 gene delivery did not affect LV systolic function post-infarction or in Ang II-mediated hypertension. Likewise, SERCA2 protein levels remained unchanged in WDR12-treated hearts post-infarction and in Ang II-mediated hypertension, whereas WDR12-induced impairment of LV systolic function was associated with the reduction of SERCA2 expressions in normal hearts. Collectively these results using different experimental animal models indicate that myocardial WDR12 modulates cardiac function through context- and disease-dependent mechanisms. The mechanisms for these distinct cardiac actions of WDR12 remain to be established, but may be due to diverse pathophysiological processes in MI and hypertension-induced heart disease [2,3]. Nonetheless, these context-dependent effects appear not to be unique for WDR12. For example, BNP [27] and p38 MAPK [13,14] gene transfers under identical experimental conditions showed distinct context-dependent effects on cardiac function and structure.

Because WDR12 gene delivery resulted in consistent elevation of BOP1 protein levels in normal adult rat hearts and post-infarction, and in Ang II-induced hypertension, but Pes1 protein levels were unaltered or increased only slightly, BOP1 may mainly mediate the distinct effects of WDR12 on cardiac function. BOP1 has a crucial role in PeBoW homeostasis and increased levels of BOP1 induces BOP1/WDR12 and BOP1/Pes1 subcomplexes and the assembly of the PeBoW complex is highly sensitive to changes of BOP1 protein levels [10]. Moreover, loss of WDR12 has no detectable effect on Pes1 expression levels, whereas reduction of BOP1 destabilized Pes1, thus showing that the interaction of Pes1 and WDR12 may be indirect and mediated by BOP1 [10]. Therefore, one possibility is that Pes1 protein expression remained largely unchanged after WDR12 gene delivery due to this indirect interaction between Pes1 and WDR12. Of note, an earlier study has reported that enforced expression of HA-tagged BOP1 was partially compensated by a decrease in endogenous BOP1, thus indicating that endogenous BOP1 expression within a cell is highly controlled [10]. Taking this into account, it might be that endogenous WDR12 expression was also partially decreased after WDR12 gene delivery.

The adverse effects of WDR12 gene delivery on LV systolic function in normal rat hearts were associated with the decreased cellular proliferation. Double immunofluorescence staining showed that proliferating cells were cardiac fibroblasts. As discussed above, PeBoW complex is essential for cell proliferation and maturation of the large ribosomal subunit [9,10,40,41]. It has been reported that depletion of endogenous WDR12 inhibited cell proliferation, suggesting that inhibition of pre-rRNA processing is coupled with repression of cell proliferation [9]. On the other hand, *in vitro* overexpression of BOP1 but not WDR12 and Pes1 inhibited cell proliferation and rRNA processing [10]. It is also notable that BOP1 seems to be a part of larger ribosome-associated complex than PeBoW containing 9 or more subunits including GATA transcription factors activator GNL3 [47] and that the integrity of the PeBOW complex is tightly controlled by protein-protein interactions and highly sensitive to elevated levels of BOP1 [10]. Therefore, we hypothesize that in our study WDR12 interaction with BOP1 resulted in decreased fibroblast proliferation, leading to thinning of LV wall, LV dilatation and LV cardiac dysfunction. Nevertheless, in view of the context-dependent actions, it is fundamental in further studies to decipher the protein complexes performing many of the diverse activities of WDR12 to cell homeostasis and proliferation.

Strikingly, WDR12 gene delivery also affected LV diastolic function, as reflected by significantly decreased E/A ratio post-infarction. In agreement with this, in human subjects the MI associated rs6725887 WDR12 variant was associated with LV diastolic dysfunction and increased left atrial size, suggesting that WDR12 is of importance for LV diastolic function also in humans. The fact that WDR12 gene transfer adversely affected LV diastolic function in the rat heart and that WDR12 was up-regulated in failing human hearts suggests that the MI associated allele of WDR12 leads to up-regulation of WDR12 expression and diastolic dysfunction in humans. As the human MI associated allele does not alter the predicted WDR12 amino acid sequence, the gene variant (or a nearby SNP in linkage disequilibrium with it) most likely exerts its effects through altered WDR12 expression levels. Further, although LV diastolic dysfunction is generally thought to be a result of rather than a risk factor for CAD, their strong co-occurrence [48] suggests that genetically WDR12 mediated risk of LV diastolic dysfunction may partially explain why WDR12 gene variance is associated with MI. Interestingly, although WDR12 genetic variance is strongly associated with MI, the association with diastolic dysfunction was independent of previous MI. Still, despite being statistically independent, we cannot exclude that the association we observe in humans could be a consequence of coronary heart disease, especially since knock-down of WDR12 has been shown to affect cholesterol metabolism [49]. On the other hand, no GWAS has so far identified genome-wide significant evidence for association between WDR12 variance and heart failure [50,51].

The pathogenic mechanisms underlying diastolic HF are incompletely understood, but comprise changes in LV myocardial structure, cardiomyocyte function, myofilamentary proteins and fibrosis in particular [52]. Since p38 MAPK overexpression causes cardiac fibrosis and depresses cardiac contractility [14], the activation of p38 MAPK pathway by WDR12 gene delivery may be linked to pro-fibrotic response and cardiac dysfunction (Fig 16). In addition, the decreased apoptotic cell death in the normal heart could be a consequence of HSP27 activation, as HSP27 can inhibit apoptosis caused by various stimuli [43]. Notably, while both fibroblast proliferation and the rate of apoptosis declined, proportionally the reduction in fibroblast proliferation was somewhat larger in accordance with the thinning of the LV and a tendency towards increased fibrosis by WDR12 gene delivery. It is also noteworthy that changes in LV systolic function, cell proliferation and apoptosis were context-dependent, but the activation of p38/HSP27 pathway, up-regulation of BOP1 levels and decrease in E/A ratio (or tendency for it) by WDR gene overexpression, were not. Overall, in the present study we could study structural and functional changes during the early remodeling process, because adenoviral gene delivery results in transient increase in WDR12 expression. Therefore, to study the longer-term effects on cardiac structure, function and mortality, detailed experiments in other models are needed.

Despite optimal treatment with the existing drugs, the prognosis of systolic HF remains poor. Moreover, to date, there is no proven effective therapy specifically for diastolic HF. Approximately half of the patients with HF have diastolic dysfunction with preserved LVEF [53]. The prevalence of HF with preserved LVEF in the community is increasing and is associated with an exceedingly high mortality [54]. Our present results indicate that local WDR12 gene delivery in rats has adverse effects on systolic and diastolic function. In particular, since MI associated WDR12 allele was associated with diastolic dysfunction, therapeutic strategies designed to specifically modulate WDR12 might represent a promising new approach to treat patients with diastolic HF.

## Limitations

A limitation of adenovirus-mediated gene delivery is that adenovirus infection may lead to a dose-dependent inflammatory reaction followed by cell death [55]. Hence, the virus dose needs to be optimized to maintain normal cell function and survival. In our earlier study under identical experimental conditions, the number of infiltrating cells in adenoviral-injected hearts was similar to that in LacZ-treated normal hearts and in hearts post-infarction [27]. Another potential limitation is that adenovirus specific and neutralizing antibodies might be generated against the transgene product [55].
